# Golgi organization regulates stem cell function in the small intestine

**DOI:** 10.1038/s41467-026-75679-1

**Published:** 2026-07-29

**Authors:** Ilya Belevich, Lucas Porcile, Agustin Sola-Carvajal, David Grommisch, Karl Annusvar, Paul Heinz, Srustidhar Das, Anna T. Webb, Simon Andersson, Nalle Pentinmikko, Eduardo J. Villablanca, James R. Goldenring, Maria Kasper, Eija Jokitalo, Robert J. Coffey, Pekka Katajisto, Sandra Scharaw

**Affiliations:** 1 Institute of Biotechnology, Helsinki Institute of Life Science, University of Helsinki, Helsinki, Finland; 2 Department of Cell and Molecular Biology (CMB), Karolinska Institutet, Stockholm, Sweden; 3 Max Planck Institute of Molecular Cell Biology and Genetics, Dresden, Germany; 4 Division of Immunology and Allergy, Department of Medicine, Karolinska Institutet and University Hospital, Stockholm, Sweden; 5 Center of Molecular Medicine, Stockholm, Sweden; 6 Research program of Molecular and Integrative Biosciences, Faculty of Biological and Environmental Sciences, University of Helsinki, Helsinki, Finland; 7 Section of Surgical Sciences, Department of Cell and Developmental Biology, and the Epithelial Biology Center, Vanderbilt University Medical Center, Nashville, TN USA; 8 Nashville VA Medical Center, Nashville, TN USA; 9 Department of Medicine, Vanderbilt University Medical Center, Nashville, TN USA

**Keywords:** Intestinal stem cells, Golgi

## Abstract

Cell-to-cell signaling between niche and stem cells regulates tissue renewal. While the identity of many mediating factors is known, it is largely unknown whether stem cells optimize their receptiveness to niche signals according to the niche organization. Here, we show that Lgr5+ small intestinal stem cells (ISCs) regulate the morphology and orientation of their secretory apparatus to match the niche architecture, and to increase transport efficiency of niche signal receptors. ISCs orient their Golgi apparatus laterally towards Paneth cells of the epithelial niche, and divide Golgi into multiple stacks. Stem cells with multiple lateral Golgi transport stem cell receptors with a higher efficiency than cells with one single Golgi. The lateral Golgi orientation and enhanced receptor transport requires A-kinase anchor protein 9 (Akap9), and is necessary for normal renewal capacity. Moreover, reduced Akap9 in aged ISCs renders ISCs insensitive to niche-dependent modulation of Golgi stack number and transport efficiency. Our results reveal a stem cell-specific Golgi complex configuration that facilitates efficient niche signal reception and tissue renewal, which is compromised in the aged epithelium.

## Abstract (web summary)

Here the authors show that intestinal stem cells reorganize their Golgi apparatus to improve the delivery of receptors that receive signals from nearby support cells. This adaptation promotes efficient tissue renewal but declines with aging, reducing stem cell responsiveness.

## Introduction

Rapid tissue turnover of the intestine is driven by Lgr5-expressing ISCs that are laterally intercalated between Paneth cells of the niche at the base of intestinal crypts^1^. Together with other cells of the niche^2–4^, Paneth cells produce multiple ISC-regulating ligands that ISCs must recognize by trafficking their respective receptors to their plasma membrane^5^. While the identities of signal-mediating ligands and receptors are a subject of intense research, the trafficking of receptors by ISCs along the secretory pathway within their three-dimensional epithelial context has not been characterized. Consequently, whether receptor trafficking provides a previously overlooked and particularly rapid optimization mechanism to control niche-to-stem cell communication, and thus tissue renewal regulation, remains unknown.

The secretory pathway is evolutionarily highly conserved and ensures the movement of newly synthesized proteins from the endoplasmic reticulum to the Golgi complex, from where the proteins are trafficked to their final destinations. While in non-polarized mammalian cells the Golgi complex comprises a single juxtanuclear stack, polarized neurons intriguingly harbor additional dendritic “Golgi outposts” in order to mediate local and fast signal delivery to their site of action^6–8^. The arrangement of organelles and polarized trafficking are intricately linked processes in polarized cells^7,9,10^. Proteins packaged at the trans-Golgi can traffic to the apical or basolateral membrane directly or via sorting endosomes, with precise regulation ensuring accurate membrane targeting^11–13^. Historically, much of the polarized trafficking work along the Golgi complex was based on the use of epithelial cells cultured as two-dimensional monolayers as model systems. However, epithelial cells reside in a three-dimensional tissue context with specific tissue architecture and renewal states, and the Golgi organization coupled to polarized trafficking in stem cells of self-renewing tissue systems or in organotypic culture is unexplored.

Beyond ensuring delivery of proteins to the cell surface, trafficking dynamics control the intensity of autocrine and paracrine signaling responses, with critical effects on cell identity and behavior. In rapidly renewing tissues such as the intestine, stem cells must continuously integrate niche-derived cues while maintaining precise control over proliferation and differentiation. Therefore, spatiotemporal control over trafficking mechanisms, influenced by subcellular Golgi positioning, has the potential to influence stem cell function in physiology and disease conditions.

Among the processes that affect tissue function, increasing the risk of developing diseases, aging plays a major role. In the small intestine, aging is associated with tissue renewal decline, morphological changes, altered motility, decreased absorptive capacity, and dysbiosis. Exhaustion of stem cell function and of cell-cell communication are mechanistic hallmarks of aging, and in the intestine, stem signaling with Paneth cells changes with age^14–16^. However, while aging-associated tissue renewal decline has been mechanistically primarily attributed to altered signaling pathways of stem cells with age^17^, whether the Golgi complex is altered, impacting trafficking of signals, remains elusive.

To address this knowledge gap, we investigated the Golgi organization of stem cells within their intestinal stem cell niche in three-dimensional organoids, examining the molecular mechanism driving Golgi localization and how this is altered in aged cells. We performed tissue volume electron microscopy, combined with microscopy-based functional assays to follow receptor transport efficiency in stem cells of the intestinal epithelium. We find that Lgr5⁺ ISCs display an adapted Golgi morphology and orientation to their epithelial niche architecture, with several Golgi stacks oriented laterally toward their niche Paneth cells to enhance polarized trafficking efficiency of niche signal receptors such as Egfr and Lgr5. Performing gene depletion and microtubule nucleation assays, we show that the A-kinase anchor protein 9 (Akap9), involved in the nucleation of microtubules from the Golgi, is essential to maintain the lateral Golgi configuration. Importantly, the peculiar Golgi organization observed in young ISCs is compromised in aged ISCs, which is associated with impaired signal responsiveness and renewal capacity.

## Results

### Intestinal stem cells surrounded by Paneth niche cells harbor lateral Golgi stacks

Proteins to be secreted migrate through the secretory pathway, where the cargo proteins are sorted at the Golgi complex according to their targeting signals^11^. In epithelial cells, the Golgi is typically localized juxtanuclear, with the trans-face oriented towards the apical cell surface^11^. Correspondingly, in the intestinal crypts, where Lgr5+ ISCs divide to produce Transient Amplifying (TA) progenitor cells that lose Paneth cell contacts and exit the niche to differentiate^1,18^, we found TA cells at the crypt neck to harbor compact juxtanuclear and apically oriented Golgi morphology (Supplementary Fig. 1a–c). However, cells at the crypt bottom displayed a strikingly lateral Golgi complex morphology (Supplementary Fig. 1a–c), whereas localization of the endoplasmic reticulum (ER) marker and cell polarization markers indicated no obvious differences between cells along the full crypt length (Supplementary Fig. 1a, d). Importantly, the extended lateral Golgi morphology coincided with the region harboring ISCs and Paneth cells, which share a particularly large lateral plasma membrane interface aiding their interactions^16^. Moreover, similar lateral Golgi morphology was also observed in the columnar ISCs of the colon (Supplementary Fig. 1e).

**Fig. 1:**
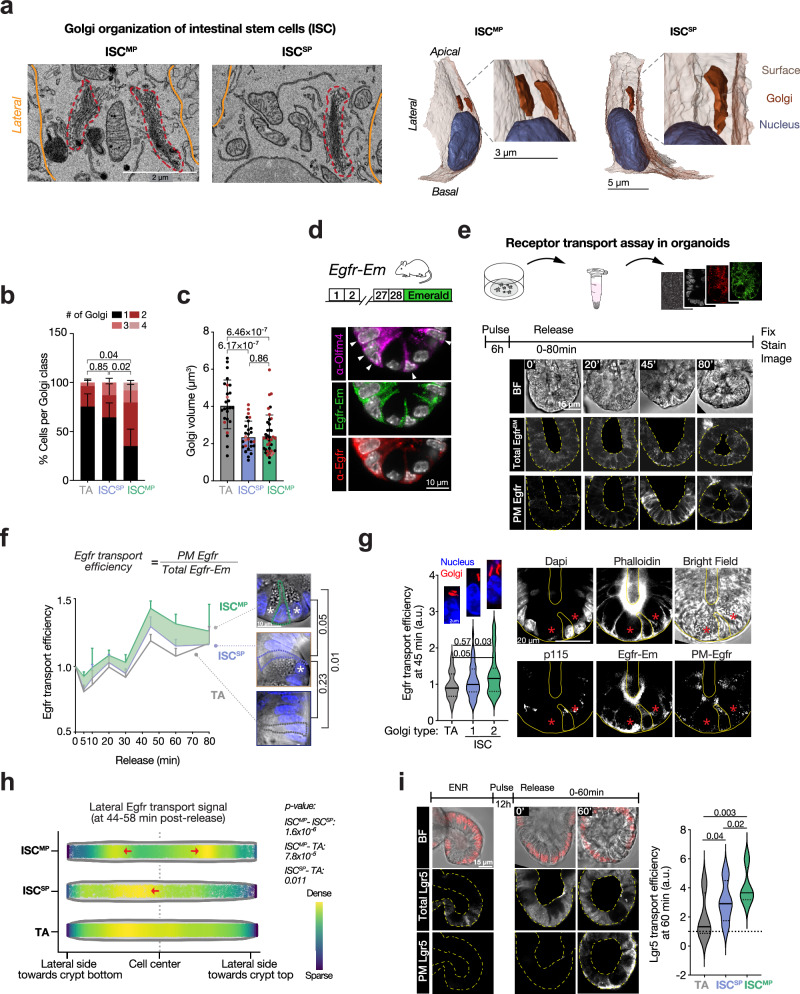
Golgi complex morphology of intestinal stem cells ensures efficient receptor transport. **a** Representative Volume EM images (left) and modeled ISC Golgi morphology (right) with **b** Golgi complex number and **c** total Golgi volume quantification (n = Golgi volume of 26 TA cells, 27 ISC^SP^ cells, and 39 ISC^MP^ cells modelled from Volume EM images of intestinal crypts obtained from 3 biologically independent mice). ISCs contacting multiple Paneth cells (PC) are referred to as ISC^MP^, those contacting a single Paneth cell as ISC^SP^, and transient amplifying progenitor cells as TA. **d** Immunostaining of Egfr-Emerald (Em) mouse crypts. Arrowheads point to Olfm4-positive ISCs. **e** Egfr-Em transport of organoid crypt cells after transport release shows maximal plasma membrane (PM) Egfr levels at 45 min post-release. BF indicates bright-field images. **f** Egfr transport efficiency quantification of organoid crypt cells. Asterisks mark Paneth cells. Data points represent means ± s.e.m. P values compare Egfr transport efficiency dynamics given by the area under the curve (*n* = 5 mice) with a two-tailed paired Student’s t-test. **g** Egfr transport efficiency of organoid crypt cells at 45 min post-release, co-stained with p115 (Golgi) and phalloidin (plasma membrane) (*n* = 3 mice). **h** Lateral Egfr-Em live-cell signal transport tracking in organoids at 44-58 min post-release. P values compare Egfr tracked intensities of organoid crypt cells along the lateral direction from 4 biological replicates (*n* = 4 mice) using a two-sided Mann-Whitney U test with Benjamini–Hochberg false discovery rate (FDR) correction to account for multiple testing. **i** Lgr5 transport efficiency quantification of organoid crypt cells. Unless otherwise mentioned, all data are represented as mean ± s.d. and compared by two-tailed unpaired Student’s t-test. *P* < 0.05 is considered significant.

To analyze Golgi complex morphologies at ultrastructural resolution and in specific cell types, we performed serial block-face scanning electron microscopy of entire intestinal crypts within intestinal tissue and rendered three-dimensional models of the cell surfaces, nuclei, and Golgi (Supplementary Fig. 1f–i; Supplementary Movie 1). Paneth cells typically displayed a single, particularly large Golgi apparatus (average volume 24 μm^3^) (Supplementary Fig. 1j). We looked at ISCs touching a single Paneth cell at the crypt edge (ISC^SP^) and at ISCs touching multiple Paneth cells at the crypt bottom (ISC^MP^). Strikingly, ISCs touching a single Paneth cell at the crypt edge typically had only one Golgi complex laterally positioned towards the contacted Paneth cell surface (Fig. 1a, b). Instead, ISCs touching multiple Paneth cells harbored multiple lateral Golgi compartments parallel oriented towards those Paneth cell neighbors that shared the largest surface area with the ISC (Fig. 1a, b and Supplementary Fig. 1k, l; Supplementary Movie 2). Moreover, the total volume of Golgi stacks in ISCs was independent of the number of Paneth cell contacts (average volume 2.4 ± 1.1 μm^3^), indicating that Golgi in ISCs contacting multiple Paneth cells is split into multiple smaller compartments (Fig. 1c). In sum, ISCs orient their Golgi apparatus laterally towards Paneth cells of their epithelial niche, and divide it into multiple compartments according to Paneth contacts.

### Lateral stem cell Golgi complexes mediate efficient stem cell receptor transport towards the surrounding niche

Unlike a central Golgi apparatus near the nucleus, neurons possess Golgi outposts positioned in the cell periphery, which increase the efficiency of localized protein secretion^7^. Thus, we asked if Golgi splitting also increases the efficiency of receptor transport in ISCs. To functionally assess ISC receptor transport dynamics, we utilized live-organoids and focused on epidermal growth factor receptor (Egfr) signaling. Egf-receptor showed a clear and gradual increase in expression from Paneth cells to TAs and ISCs, which was specific to Egfr among the Egf receptor family members (Supplementary Fig. 2a; Supplementary Data 1 and Supplementary Tables 1–2). To visualize Egfr transport of ISCs in real-time in organoids, we used a mouse model expressing endogenous Egfr-Emerald fusion protein (Egfr-Em)^19^. Intestines from these mice demonstrated normal intestinal morphology and organoid growth (Supplementary Fig. 2b, c), and exhibited high Egfr enrichment in Olmf4+ ISCs as expected (Fig. 1d).

We used a previously established approach^20^, which we applied to organoids, to induce de novo Egfr-Em synthesis and accumulation at the ER and to follow its trafficking to the plasma membrane (PM) (see Methods, Supplementary Fig. 2d–g). Specifically, we depleted Egfr from the cell surface of organoid cells by inducing rapid internalization with a supraphysiological long-term pulse of Egf (200 ng/ml) (ENR^HighE^ medium) (Supplementary Fig. 2d, e). The Egf pulse-induced depletion of Egfr from the plasma membrane induced the accumulation of newly synthesized Egfr-Em in the ER (Supplementary Fig. 2f). In line with previous reports^21,22^, prolonged Egf also induced expression of Lrig1 and TGFα (Supplementary Fig. 2g). Change into Egf-free media (NR) after ENR^HighE^ pulse (“release”) increased PM-Egfr due to trafficking of the newly synthesized Egfr (Supplementary Fig. 2h, i), and Egfr transport efficiency could be addressed by following the increase in ratio between plasma membrane associated Egfr (PM-Egfr, detected by an antibody recognizing the extracellular domain of Egfr) and total Egfr of the cell (detected by Egfr-Em intensity)^20,23^.

We found that the transport efficiency of Egfr from the ER to the surface reached its maximum after 45 minutes (Fig. 1e and Supplementary Fig. 2h–j). This transport rate was similar to other cargo proteins^24,25^, and PM-Egfr increase was specific to Egf pulse-induced Egfr synthesis and blocked by the secretion inhibitor Brefeldin A^26^ (Supplementary Fig. 2k).

As Paneth cells are one source of Egf ligands in the intestine, epithelial organoids devoid of non-epithelial cells provide a simplified system to address possible relationships between niche organization and Egfr transport efficiency. We therefore analyzed Egfr transport efficiency in organoids, separately for ISC^MP^, ISC^SP^ and TA cells, and for ISCs containing either one or multiple Golgi. Strikingly, ISC^MP^ transported Egfr more efficiently than ISC^SP^ and TA cells (Fig. 1f), with a difference in efficiency similar to biologically relevant outcomes in other systems^27^. Similarly, ISCs with multiple lateral Golgi compartments transported Egfr more efficiently than ISCs with one lateral Golgi (Fig. 1g), corroborating that the organization of the ISC Golgi and the organization of the niche are functionally linked. Further, tracking of individual transport events revealed that the more efficient Egfr transport in ISC^MP^ occurs towards the Paneth cells multi-laterally, whereas ISC^SP^ favors transport uni-laterally directed towards the one Paneth cell they contact (Fig. 1h, red arrows).

We next established a transport assay for the leucine-rich repeat-containing G protein-coupled receptor Lgr5, whose R-spondin ligands are provided basally to amplify Wnt signaling^28^. Similar to Egfr synthesis induction, we also induced de novo Lgr5 synthesis (See Methods, Supplementary Fig. 2l) using a prolonged 1000 ng/ml R-spondin (ENR^HighR^) pulse. This allowed us to follow the increase in the ratio between plasma membrane-associated Lgr5 (PM-Lgr5, detected by an antibody targeting the extracellular domain of Lgr5^29^) and total cellular Lgr5 (detected by an antibody recognizing the intracellular domain of Lgr5). We found that ISC^MP^ transport Lgr5 receptor more efficiently than ISC^SP^ and TA cells, indicating that the multiple Golgi compartments of ISC^MP^ increase the overall transport of receptors in response to the provided key niche signals (Fig. 1i).

In sum, ISCs harbor multiple lateral Golgi compartments that facilitate efficient transport of key receptors according to the availability of their stemness-maintaining extracellular niche cues.

### Lateral Golgi localization requires Akap9

**Fig. 2:**
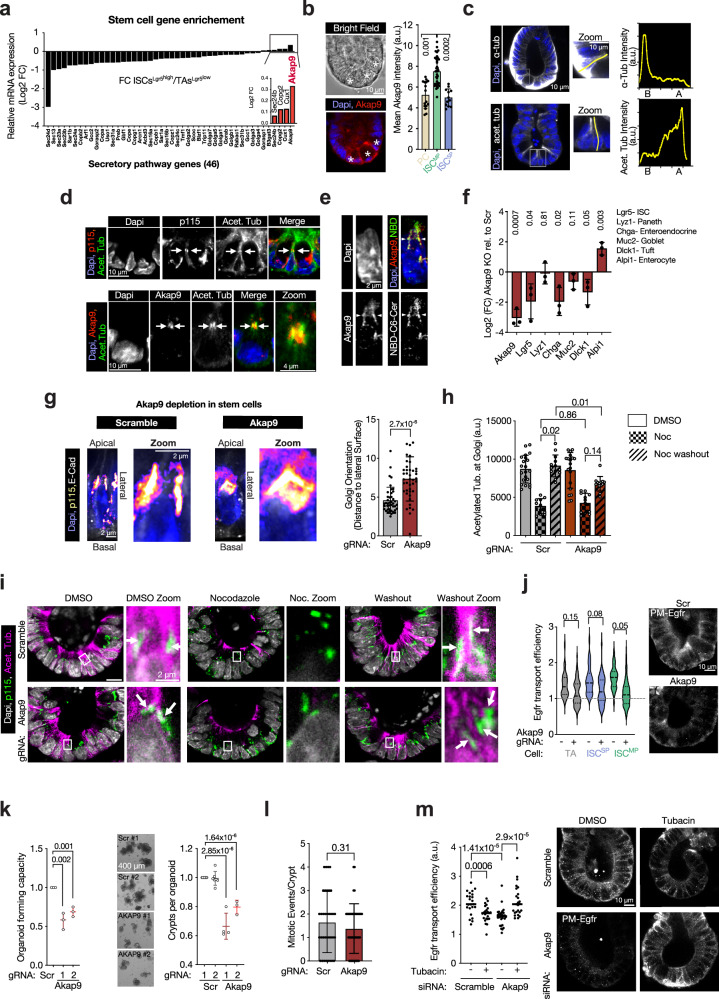
Golgi complex morphology is regulated by Akap9. **a** RNA-expression enrichment analysis of FACS-sorted intestinal stem cells (ISCs) vs. transient amplifying (TA) progenitors. **b** Immunostaining of Akap9 in small intestinal organoids. Asterisks mark Paneth cells (PCs) (*n* = 16 Paneth cells, 35 ISCs contacting multiple Paneth cells (ISC^MP^), and 12 ISCs contacting a single Paneth cell (ISC^SP^) analyzed from organoids derived from 3 biologically independent mice). **c** Organoid immunostaining shows lateral-elongated enrichment of acetylated tubulin towards the apical (A) region and of alpha-tubulin in the basal (B) region (*n* = 3 mice). **d** Acetylated tubulin and Akap9 colocalization on p115-positive ISC Golgi stacks in intestinal organoids. **e** Akap9 and Golgi NBD-C6-Ceramide-stained colocalization within ISCs of organoids. ISCs were identified by their columnar shape and crypt base localization intercalated between PCs with flattened nuclei and apical granules (*n* = 3 mice). **f** RT-qPCR analysis of Akap9 CRISPR-depleted (KO) vs. Scramble (Scr) control organoid lysates (*n* = 3). **g** Golgi orientation analysis in relation to the lateral surface in Akap9 vs. Scr CRISPR-targeted ISCs (*n* = 3 mice). **h** Quantification and **i** representative images of acetylated tubulin re-nucleation at p115-positive Golgi complex stacks of crypt bottom ISCs in Akap9 CRISPR-depleted vs. Scr-targeted control organoids. Organoids were treated for 1 h with 10 µM Nocodazole, followed by a 2 h washout. Each data point in (h) represents the mean measured intensity of acetylated tubulin at p115-positive Golgi complexes of ISCs per organoid. (*n* = 3 independent experiments with two-tailed unpaired Student’s t-test comparing organoid averages per experiment). **j** Egfr transport efficiency at 45 min post-release compared to no-release (grey line) in Akap9 vs. Scr-targeted organoid crypt cells. Data represent the transport efficiency of single cells from 10 organoids per biological replicate (*n* = 3 mice/condition). Conditions were compared by two-tailed paired Student’s t-test between the mean values. **k** Organoid forming capacity and regenerative growth of Akap9 CRISPR-depleted vs. Scr-targeted organoids. *N* = 3 independent CRISPR-generated organoid lines derived from 3 biologically independent mice (left graph). *N* = 3-7 independent CRISPR-generated organoid lines derived from 3-7 biologically independent mice (right graph). Each dot represents the mean of 30–200 organoids per line. **l** Crypt domains of Akap9 CRISPR-depleted vs. Scr-targeted organoids were analyzed for the number of nuclei in cell division per single imaging z-plane in organoids. Nuclei were identified based on their Dapi signal and cell surface outlines based on bright-field images. Mitotic cells were scored as apically protruded cells with dividing nuclei (*n* = 35 Scr and 42 Akap9 CRISPR-depleted organoids collected across 4 independent experimental repeats). **m** Egfr transport efficiency at 45 min post-release in Akap9 mRNA-downregulated vs. Scr-targeted control organoids treated for 2 h with 10 µM Tubacin vs. DMSO control. Data represent the mean transport efficiency of single ISCs per organoid (*n* = 23, 24, 29, and 25 organoids for Scr + DMSO, Scr + Tubacin, Akap9 + DMSO, and Akap9 + Tubacin, respectively). Organoids were derived from 3 biologically independent mice, which served as the biological replicates, with two-tailed unpaired Student’s t-test comparing the organoids. Unless otherwise mentioned, conditions are represented as means ± s.d. and were compared by two-tailed unpaired Student’s t-test. *P* < 0.05 is considered significant.

We next aimed to identify key genes controlling Golgi morphology and organization in ISCs. To this end, we analyzed RNA sequencing data of sorted Paneth cells, ISCs (Lgr5^high^) and TA (Lgr5^low^) cells^15,16^ for known secretory pathway machinery genes (Supplementary Table 3), including Golgi, COPI and COPII vesicular carrier genes. While the expression of most genes was highest in the secretory Paneth cells and intermediate in TA cells, four genes were expressed the highest in ISCs (Fig. 2a and Supplementary Tables 3–5). The strongest enrichment was noted for the A-kinase anchor protein 9 (Akap9) gene (also known as AKAP350, AKAP450 or CG-NAP)^30–32^, which is directly linked with Golgi function and morphology^30,33^. Interestingly, Akap9 localizes to the cis-Golgi, where it initiates nucleation of microtubules and maintains microtubule acetylation^34,35^ for example, in migrating cells and Golgi outposts of neurons^34,36,37^. Consequently, Akap9 regulates Golgi localization^33^. We found that Akap9 protein level is high specifically in ISCs with multiple Paneth cell contacts (Fig. 2b), and Akap9 colocalized with acetylated tubulin at lateral Golgi of ISCs (Fig. 2c–e and Supplementary Fig. 3a).

To investigate the functional relevance of Akap9 for Golgi organization in organoids we depleted Akap9 with lentiviral CRISPR-targeting (Fig. 2f). We noted a collapse of lateral Golgi complexes in Akap9-targeted organoids, resulting in apically oriented Golgi morphology also in cells between Paneth cells and expected to represent stem cells (Fig. 2g). To investigate whether the effect of changed Golgi morphology upon Akap9 depletion was linked to its role in nucleating microtubules at Golgi membranes, we performed a nocodazole washout assay and specifically quantified acetylated tubulin within the Golgi region of crypt bottom ISCs of Akap9 CRISPR-depleted organoids and as control scrambled-targeted organoids. We observed that following 1-h nocodazole treatment, acetylated tubulin levels were similarly reduced in both control and Akap9-depleted cells (Fig. 2h, i). Upon nocodazole washout, control cells showed robust microtubule re-establishment at the Golgi, whereas Akap9-depleted cells displayed only partial recovery (Fig. 2h, i). These results provide direct functional evidence that Akap9 is required for efficient microtubule nucleation at Golgi membranes of ISCs and provide a mechanistic explanation for the loss of lateral Golgi polarization under Akap9 depletion. Interestingly, targeted organoids showed reduced expression of stem and enteroendocrine markers, while the enterocyte marker Alpi was increased, suggesting that Golgi alterations had influenced cell fate choices and stem cell maintenance (See Fig. 2f). To address the role of Akap9-mediated lateral Golgi organization for receptor transport while maintaining Akap9 function^38^, we selected organoid clones with a reduction in Akap9 mRNA levels comparable to the difference in Akap9 expression between ISCs and TA cells (Supplementary Fig. 3b). Akap9 targeting reduced ISC^MP^ Egfr transport efficiency, abrogating cell type-specific differences (Fig. 2j). Finally, stem cell-dependent colony-forming capacity and regenerative growth of organoids were significantly reduced upon Akap9 depletion (Fig. 2k), despite normal mitosis rates (Fig. 2l). Jointly, these data suggest that Akap9 regulates stem cell-mediated tissue renewal by enabling the ISC-specific Golgi morphology and high transport efficiency. Next, we addressed whether lateral Golgi organization is a requirement for increased secretory trafficking in ISCs. To achieve this, we performed Akap9 siRNA-mediated knockdown in intestinal organoid cells (Supplementary Fig. 3c). We then pharmacologically rescued Golgi microtubule acetylation using Tubacin, a selective HDAC6 inhibitor that increases microtubule acetylation^39^, and measured Egfr trafficking. As expected, Tubacin treatment increased acetylated microtubules at the Golgi complexes in both scrambled control and Akap9 knockdown ISCs of organoids (Supplementary Fig. 3d, e). Notably, Tubacin treatment alone reduced Egfr transport efficiency in control cells, indicating that perturbation of microtubule acetylation is sufficient to disrupt this process (Fig. 2m). Importantly, lateral Golgi positioning and Egfr transport efficiency, which were both impaired in Akap9 knockdown ISCs, were restored upon Tubacin treatment (Supplementary Figs. 3f, g and 2m). These results indicate that Golgi-associated acetylated microtubule organization itself can compensate for Akap9 loss and is required for efficient receptor trafficking, providing direct functional evidence that supports the requirement of lateral Golgi organization in efficient stem cell receptor trafficking. In line, ligand-receptor-independent induction of stem cells in organoids with CHIR99021 and valproic acid^40^ which lack mature Paneth cell niche contacts, resulted in reduced Akap9 expression (Supplementary Fig. 3h) and in the increase of Lgr5+ stem cells with a collapsed Golgi (Supplementary Fig. 3i). We therefore conclude that the niche signals guide the organization of Golgi into lateral units (See Fig. 1a, b), which is integral to stem cell identity.

### Stem cell receptor transport is compromised during aging

**Fig. 3:**
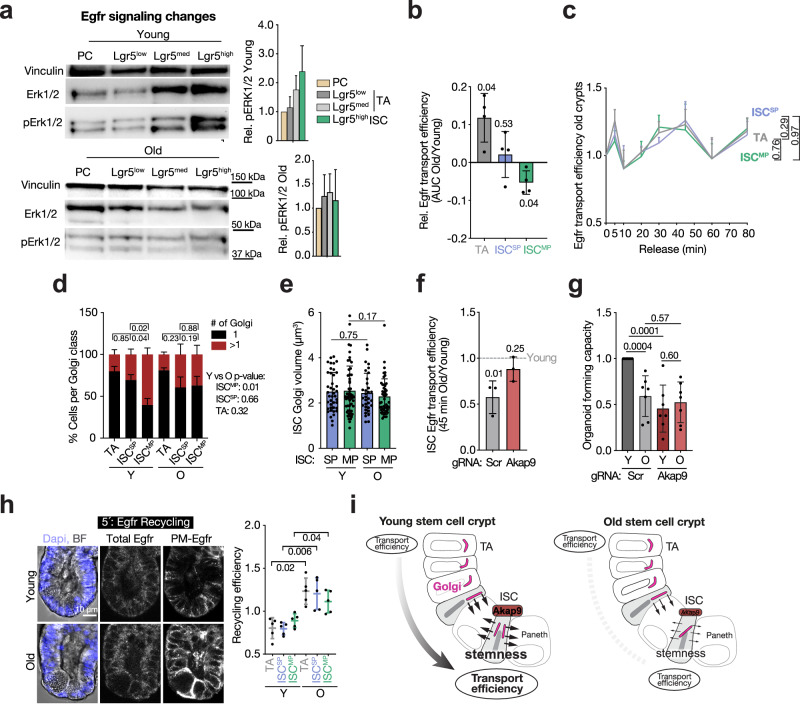
Altered Golgi morphology correlates with decreased Egfr transport in old stem cells. **a** Immunoblots and quantification of FACS-sorted intestinal stem cells (ISCs) (Lgr5^high^), early and late transient amplifying (TA) progenitors (Lgr5^med^ and Lgr5^low^) and Paneth cells (PCs) (*n* = 3 biologically independent mice per age group. For each sample, an equal number of cells (50,000–100,000) per population were sorted for downstream analysis). **b** Egfr transport efficiency between 0–80 min post-release compared by the area under the curves (AUC) in Egfr-Em organoid crypts from old vs. young mice (*n* = 4 mice). **c** Egfr transport efficiency in organoid crypts of old mice. Data points represent means ± s.e.m. and cell types are compared by two-tailed paired Student’s t-test of the AUC (*n* = 4 mice). **d** Golgi stack number quantification based on volume EM imaging (crypts from *n* = 5 young mice, *n* = 3 old mice). P-values were calculated based on the proportions of cells per crypt containing 1/ > 1 Golgi complex with a two-way ANOVA test. **e** Young: *n* = 41 ISC^SP^ cells (touching a single Paneth cell) and 54 ISC^MP^ cells (touching multiple Paneth cells) analyzed from EM-based reconstructions of intestinal crypt cells obtained from 4 biologically independent mice. Old: *n* = 33 ISC^SP^ cells and 48 ISC^MP^ cells analyzed from EM-based reconstructions of intestinal crypt cells obtained from 3 biologically independent mice. **f** *n* = 103 old Scr ISCs (compared to 104 young Scr ISCs), 98 old Akap9-depleted ISCs (compared to 99 young Akap9-depleted ISCs) were analyzed from 10 organoids per condition. Organoids were generated from 3 independent CRISPR-generated organoid lines derived from three biologically independent mice. Data points represent the mean ± SD of the ISC measurements for each experimental condition. **g** Organoid forming capacity (*n* = 5 mice). **h** Egfr recycling efficiency in organoid crypts. Conditions are compared by two-tailed paired Student’s t-test (*n* = 5 young, *n* = 5 old mice). **i** Schematic model of the proposed ISC mechanism to regulate receptor transport efficiency through Golgi organization. Unless otherwise mentioned, data are represented as mean ± s.d. and conditions are compared by two-tailed unpaired Student’s t-test. *P* < 0.05 is considered significant.

Impaired stem cell function and intercellular communication are hallmarks of aging^14^. This ultimately leads to decreased tissue turnover and aging-associated complications in many tissues, including the intestine^41,42^. Interestingly, we found that aging reduces Akap9 expression in ISCs (Supplementary Tables 6, 7; Supplementary Fig. 3j). Moreover, Egfr pathway activation marked by Erk1/2 phosphorylation was dramatically reduced and cell type-specific differences were lost in the cells from old compared with young Lgr5-eGFP-IRES-reERT2 reporter mice^1^ (Fig. 3a and Supplementary Fig. 3k). We therefore assessed whether receptor transport efficiency is altered in old ISCs, potentially contributing to the decline in intestinal renewal capacity during aging^15,43^. Indeed, aging reduced Egfr transport efficiency, particularly in the ISC^MP^ (Fig. 3b and Supplementary Fig. 3l left panel), rendering all proliferating cell types equally capable in Egfr transport (Fig. 3c, compared to young in Fig. 1f). This prompted us to investigate whether Golgi morphology is altered in old ISCs. Strikingly, the multiple Golgi complexes that we observed in the majority of young ISC^MP^ were lost in old crypts, and the majority of old ISCs - irrespective of their Paneth cell contacts - contained only one Golgi complex (Fig. 3d). However, total Golgi volume per cell was not changed in old stem cells (Fig. 3e), suggesting that the age-induced change in Egfr transport is associated with alterations in Akap9-mediated lateral Golgi organization rather than size. Supporting this notion, Akap9 depletion had no effect on Egfr transport in old ISCs, making them as defective as young ISCs after Akap9 reduction (Fig. 3f), and Akap9 depletion did not further reduce organoid-forming capacity of old ISCs (Fig. 3g).

In addition to total transport efficiency, also the temporal regulation of Egfr transport was changed in old crypt cells, with more Egfr-Em reaching the plasma membrane as early as 5 min after release compared to young organoid crypt cells (Fig. 3h and Supplementary Fig. 3l). Colocalization with recycling Apo-transferrin^44^ (Supplementary Fig. 3m) and blockage by the Egfr recycling inhibitor primaquine^45,46^ (Supplementary Fig. 3n), together demonstrated that recycling of Egfr is increased in old intestinal epithelium, while delivery of newly produced Egfr via the Golgi to the cell surface is reduced particularly in stem cells contacting multiple Paneth cells (See Supplementary Fig. 3l left panel). Taken together, our findings suggest that during aging, the inability to match Golgi organization with niche contacts correlates with reduced Egfr transport efficiency, but such defects are partly compensated by increased receptor recycling at the plasma membrane.

## Discussion

The precise spatial and temporal regulation of cell-to-cell signaling is essential for cell fate control, exemplified by alterations in the key stem cell-regulating Egf and Wnt pathways during aging^15,47^. However, whether such alterations result from a reduction in the availability of ligands or from the reduced ability of stem cells to receive such vital niche signals has been challenging to address. We discovered that stem cells modify the central organelle of the secretory pathway, the Golgi complex, according to the architecture of the niche, and thereby appear to customize their signal-receiving ability, matching it with their own specific niche. Our data collectively suggest that the Akap9-regulated lateral Golgi orientation of ISCs is a stem cell-intrinsic ability that allows the niche-dependent Golgi remodeling for rapid and polarized stem cell signal trafficking.

We studied the Golgi complex in the intestinal epithelium, where ISCs at the edge of the niche, contacting one Paneth cell, have a lower likelihood to originate a long-term clone than ISCs at the center of the niche, which contact multiple Paneth cells^48,49^. As one-sided niche signals induce differentiation of embryonic stem cells, whereas multi-sided signals maintain self-renewal^50^, it is possible that optimization of niche signal receptiveness that is facilitated by Golgi orientation is an important step in many systems. The Golgi complex was found to reorient to the leading edge during directed cell migration towards a wound^51,52^ and during T-cell detection of target cells^53,54^. The stem cell Golgi complex regulator identified here, Akap9, is deregulated in Alzheimer's disease^55,56^, and various cancer types^57–59^ leading to promoted cell migration^57,59^. The niche-dependent Golgi plasticity discovered here may therefore provide insights into multiple multicellular systems where heterotypic cell-cell interactions are critical.

Molecular mechanisms guiding the Golgi “splitting” were not revealed by our studies, but it is possible that cues from Paneth cells that support stem cell identity and regulate cell polarization, including Delta-like^60,61^ and Wnt ligands^2^, serve also as signals guiding Golgi organization and thereby orienting stem cell receptiveness. Nevertheless, the finding that restoring microtubule acetylation significantly restored lateral Golgi polarization and trafficking suggests that Golgi-associated microtubule organization is a key determinant of efficient trafficking from the lateral ISC Golgi complexes and that it can be modulated downstream of Akap9.

While enriched in ISCs, the scaffolding protein Akap9 is also expressed at relatively low levels in TA and Paneth cells. High Akap9 expression in ISCs may exceed a functional threshold to enable Golgi-based microtubule nucleation and multivalent scaffolding at the Golgi cisternae, pushing Golgi stacks laterally. Instead, lower Akap9 levels likely restrict its activity primarily to centrosomal roles^38^, limiting its capacity to reorganize Golgi architecture. Alternatively, Akap9 might act in concert with other proteins specific for ISCs to mediate the peculiar Golgi subcellular localization observed in these cell types.

In general, the organization and positioning of the Golgi apparatus are mediated by the microtubule cytoskeleton, which provides structural support and controls Golgi stability^62^. Disruption of microtubule organization leads to Golgi fragmentation and mislocalization, impairing polarized trafficking and signal reception. Of note, both aging and cancer have been associated with progressive defects in microtubule stability^63^, impaired centrosomal function^64^, and reduced cytoskeletal dynamics^65,66^. Such age-related microtubule impairment could thus represent an upstream driver of Golgi mislocalization in aged stem cells, impacting their ability to orient toward niche signals and disrupting signal trafficking. Cytoskeletal decline may thus represent a structural determinant of stem cell aging, linking broader cellular deterioration to altered Golgi architecture and reduced niche responsiveness.

While the molecular composition of the organelles of the secretory pathway has been intensively investigated over the last decades^67–70^, the arrangement of this endomembrane system within multicellular tissues is still not well understood and represents an unexplored organization level. Polarized cells are largely described to possess a nucleus near the basal side and the Golgi organelle above the nucleus and beneath the apical membrane^71,72^ whereas we find the Golgi complex to be organized into multiple units and polarized in accordance with the niche architecture. This demonstrates that the Golgi complex can be highly spatially organized in a cell-type- and context-dependent manner within tissues, which ultimately contributes to functional cellular diversity underlying tissue function.

Beyond the intestinal epithelium, our findings raise the possibility that subcellular localization of the Golgi complex might represent a general and previously underappreciated regulator of stem cell function. If Golgi positioning can control the efficiency and polarity of signal cues, other stem cells may exploit this mechanism to rapidly respond to dynamic environmental conditions without requiring transcriptional reprogramming. Such a mechanism would allow stem cells to integrate competing or asymmetric signals, influencing cell fate decisions and stem cell function at single-cell resolution. In aging, progressive perturbations in organelle organization might thus act as an early cellular mediator of tissue decline. More broadly, modulation of Golgi position and organization could represent a novel target to boost tissue regeneration in the aging context and limit cancer initiation. In the future, we foresee that approaches to enhance Akap9 activity in a cell-specific manner, possibly by using intestinal organoids as a high-content screening platform^73^ using Egfr signaling or Golgi positioning as readouts, could accelerate the development of therapies aimed at improving ISC function.

Limitations of the study:

Using organoids as a model, our study identifies a stem cell-intrinsic polarized Golgi organization to optimize stem cell communication with their epithelial niche, which is compromised in aged ISCs. Further mechanistic studies of Akap9-mediated Golgi organization and dynamics in the aging context require a reliable in vitro aging organoid model. While we are able to observe a reduced renewal rate in organoids from old animals in the first passages, thus mimicking the aged tissue state, we find that this characteristic cannot be maintained with long-term organoid passaging. To identify strategies to restore Golgi complex organization in the context of aging, an aged organoid model will need to be established.

In addition, while we focus on the altered Golgi complex organization of ISCs in their epithelial niche, the contribution of the stromal niche, including age-related extracellular matrix remodeling, remains to be addressed.

## Methods

### Animal housing

Lgr5-eGFP-IRES-CreERT2^1^, Egfr-Em^19^ and wild-type mice were maintained with a C57BL/6 J background and housed at Karolinska Institute and Max Planck Institute of Molecular Cell Biology and Genetics in IVC cages at a consistent temperature (19-23 °C) and humidity (55% ± 10 %) under a 12 h light–dark cycle. Standard chow and water were accessible ad libitum. Aging experiments were performed with animals of 3–6 months (referred to as “young”) and of 22 months or older (referred to as “old”), with both sexes used. Animal housing and all experimental procedures were performed in accordance with national and institutional guidelines and regulations.

### Isolation of mouse small-intestinal crypts

Small-intestinal crypts were isolated from mouse intestinal tissue^74^. Mouse small intestines were flushed with cold PBS, longitudinally opened, and the mucus was removed. The intestine was cut into approximately 0.5 cm pieces and incubated with 10 mM EDTA in PBS on ice for 2 h with changes at 10 min, 3 × 15 min and 1 h. After each incubation, tubes were gently shaken, the supernatant containing epithelium was discarded, and fresh PBS-EDTA was added. After the last incubation, the tissue suspension was vigorously shaken, and crypts were enriched by filtering through a 70-μm nylon mesh. Crypts were collected by centrifugation at 4 °C, 300 x *g* for 5 min and washed once with cold PBS.

### Intestinal organoid culture

Isolated intestinal crypts were plated (50–200 crypts per 20 μl drop of 60% Matrigel) and overlaid with ENR medium consisting of Advanced DMEM/F12 (Life Technologies) supplemented with 1 x Glutamax (Gibco), 1x Penicillin–Streptomycin (Sigma), 10 mM Hepes, 50 ng/ml mouse Egf (R&D Systems), 100 ng/ml Noggin (Peprotech), 500 ng/ml R-spondin-1 (R&D Systems), 1 x B-27 (Life Technologies), 1 x N-2 (Life Technologies) and 1 μM N-acetyl-L-cysteine (Sigma-Aldrich). 10 μM Y-27632 (Sigma) was added for the first two days of culture. The medium was replenished every two days. ENR^HighE^ contained mouse Egf (R&D Systems) at 200 ng/ml. ENR^HighR^ contained mouse R-Spondin-1 (R&D Systems) at 1000 ng/ml. The formation of clonogenic organoids (forming capacity) was counted after two days of culture, and organoids' regenerative growth (number of de novo crypt domains per organoid) after 5 days of culture. To induce stem cells in organoids^40^ (Supplementary Fig. 3i), we supplemented organoid ENR medium from day two of culture with 3 μM Chir99021 (Sigma) and 1 mM Valproic Acid (Cayman Chemicals). The treatment was then repeated every two days.

### Serial block-face scanning electron microscopy (SBF-SEM) (3D Volume EM)

#### Sample preparation

Mice were perfused with 10 ml of 0.9% NaCl (RT) followed by 15–20 ml of 2% formaldehyde, 2% sucrose in 0.1 M sodium cacodylate buffer (pH=7.4) supplemented with 2 mM CaCl_2_. 0.5–1 cm jejunal segments (6–10 cm from the stomach) were dissected out and fixed in 2% formaldehyde, 2.5% glutaraldehyde, 2% sucrose in 0.1 M sodium cacodylate buffer (pH=7.4) supplemented with 2 mM CaCl_2_ for 3 h. Initially, the specimens (5 “young” and 3 “old”) were stained with an adapted NCMIR protocol (Deerinck et al., 2022, 10.17504/protocols.io.36wgq7je5vk5/v2) where the incubation steps were aided by microwave application using a Pelco Biowave Pro+ microwave processing system (Ted Pella, Redding, CA). To improve penetration of staining agents into the tissue, the recent specimens (1 “young” and 1 “old”) were prepared using a modified Hua et al. protocol^75^, where the washing, dehydration and resin infiltration steps were performed under microwave irradiation.

In all cases, the embedding was performed in Durcupan ACM resin (Sigma-Aldrich). The blocks were then mounted on aluminum specimen pins (EM Resolutions Ltd, Sheffield, UK) using conductive silver epoxy (CircuitWorks CW2400) and trimmed in a pyramidal shape. Then, the entire surface of the specimen was sputtered with a 5-nm layer of platinum coating (Q150TS coater, Quorum Technologies, Laughton, UK) to improve conductivity and reduce charging during the sectioning process.

### SBF-SEM data acquisition

All SBF-SEM data were acquired using a Quanta 250 Field Emission Gun SEM microscope (FEI Co., Hillsboro, OR) equipped with a 3View System (Gatan Inc., Pleasanton, CA) using a backscattered electron detector (Gatan Inc., Pleasanton, CA). The imaging conditions varied depending on sample quality, with a general beam voltage of 2.5 kV, spot size 3, pressure of 0.08–0.22 Torr, and a 10 μs dwell time. After imaging, Microscopy Image Browser (MIB)^76^ was used to process, align, and segment the SBF-SEM image stacks.

### Modelling of crypt organelles

Models of organelles were generated in MIB^76^. The cell boundaries were segmented semi-automatically using the Graphcut tool of MIB, while the Golgi stacks were manually identified and segmented using the Brush tool aided by an interpolation technique to fill the gaps between slices with the drawn profiles. All nuclei (except one crypt segmented with the GraphCut tool) were segmented using a 2D DeepLabV3-Resnet50^77^ convolutional neural network trained and applied in DeepMIB^78^. The final model of the nuclei was generated by multi-view fusion, where 2D predictions from sagittal, coronal, and axial planes are fused together. Finally, the generated models were manually checked and corrected in MIB and visualized in Amira software (Thermo Fisher Scientific). Cell types were identified as follows: Paneth cells as crypt bottom cells with large granules, ISCs as in direct contact with multiple Paneth cells (ISC^MP^), ISCs with a single Paneth cell contact (ISC^SP^), and TA cells as cells at the crypt neck and in no contact with Paneth cells.

### Golgi orientation quantification

To quantify the spatial relationship between the Golgi apparatus orientation and the cell boundary in SBF-SEM and confocal microscopy datasets, a segmentation-based approach was employed. Here, the Golgi and the entire cell are first segmented to create distinct 3D objects. Subsequently, a distance map is calculated based on the cell outline, assigning each voxel within the cell to a value corresponding to its distance from the nearest boundary. Finally, the relative orientation of the Golgi apparatus to the cell boundary can be assessed by analyzing the standard deviation of the distance map values within the segmented Golgi material (Supplementary Fig. 1l). A high standard deviation would indicate a dispersed distribution of distances within the Golgi, suggesting its more perpendicular orientation relative to the cell edge. Alternatively, a low standard deviation would indicate a more parallel and thus lateral orientation of the Golgi relative to the lateral cell boundary. To streamline the analysis, we developed a plugin for MIB that is capable of processing multiple objects and generating results in Excel or MATLAB formats. This plugin is available starting from MIB version 2.93, with detailed instructions and demo datasets accessible at 10.5281/zenodo.21068508^79^.

### Egfr transport assay

Egfr is internalized and degraded upon Egf binding^80,81^, necessitating transport of newly synthesized Egfr to the plasma membrane^20,82^ to maintain niche signal responsiveness. In the intestine, Egfr ligands are produced by multiple cell types within the ISC niche^5,83^, including the ISC-neighboring Paneth cells (Supplementary Table 2), and they support stem cell function during homeostasis and regeneration^84–86^.

To visualize ISC Egfr transport in real-time, we grew organoids from Egfr-Em mice. The high Egfr enrichment in Olfm4+ ISCs ensured that ISC Egfr traffic to the plasma membrane could be scored without interference from the surrounding Paneth cells (Fig. 1d). Egfr-Em intestinal organoids were cultured in ENR media in 48-well culture dishes for 4 days. This was followed by 24 h incubation with NR medium without supplemented Egf (Supplementary Fig. 2d). In order to monitor the transport of newly synthesized Egfr-Em from the endoplasmic reticulum to the cell surface, we depleted Egfr from the cell surface by inducing rapid internalization with a supraphysiological long-term pulse of Egf (200 ng/ml)^20^ (Supplementary Fig. 2e). Organoids were then induced for new Egfr synthesis by the long-term incubation with 200 ng/ml Egf (ENR^HighE^) for a time of 6 h, referred to as “pulse”, if not otherwise indicated in the figures (Supplementary Fig. 2d–f). As a control, 200 ng/ml Pdgf (Sigma) was used in the NR medium for the pulse (Supplementary Fig. 2k).

The transport efficiency of Egfr was addressed by following the increase in the ratio between plasma membrane-associated Egfr (PM-Egfr, detected by an antibody recognizing the extracellular domain of Egfr) and total Egfr of the cell (detected by the Egfr-Emerald intensity)^20,23^ (Supplementary Fig. 2h–j). To achieve this, we induced the transport of the newly synthesized Egfr, referred to as “release”, by a washout step of the ENR^HighE^ media and replaced it with Egf-free media (NR) (Fig. 1e). For this, organoids were scraped from the culture dish and collected into 1.5 ml low-binding microcentrifuge tubes. The organoids were extracted from the Matrigel by pipetting in ice-cold PBS, followed by organoid pelleting through centrifugation at 300 x *g* for 30 s. Advanced DMEM/F12 medium was supplemented with 100 µg/ml cycloheximide (Merck) to stop new protein synthesis, and organoids were incubated for the indicated transport release times at 37 °C (Supplementary Fig. 2h–j). For the combination of the Egfr assay with brefeldin A (Sigma) (Supplementary Fig. 2k) or primaquine (Sigma) (Supplementary Fig. 3h), 5 µg/ml brefeldin A was added to the pulse 30 min before release, or 0.3 mM primaquine for 60 min before release.

After the release times, organoids were pelleted and fixed with 4% PFA for 15 min at room temperature (RT). Following fixation, organoids were blocked for 30 min at RT with 5% Normal Goat Serum (Thermo Fisher Scientific) in DPBS (Dulbecco’s PBS 0.01 M + 0.2% BSA). The organoids were then incubated with mouse anti-Egfr extracellular domain (Clone 199.12, Thermo Fisher Scientific, MA5-13319, dilution 1:40) overnight. The next day, organoids were washed 3 times in DPBS and incubated for 2 h with Alexa Fluor 647-conjugated anti-mouse secondary antibody (Thermo Fisher Scientific, A-21235, 1:1000) in blocking buffer at RT, followed again by 3x DPBS washes. Thereafter, DAPI (Life Technologies, 1 μg/ml) staining was performed for 30 min at RT. The organoids were taken up in Immu-Mount media (Thermo Fisher Scientific) and placed on a 14 mm glass-bottom MatTek dish with a coverslip on top. Coverslips were sealed with nail polish.

Images of the nucleus (DAPI), total Egfr-Em (Emerald GFP), plasma membrane Egfr (Alexa Fluor 647) and bright-field images were acquired by confocal microscopy. Using ImageJ, single cells of imaged organoids were segmented, and their transport efficiency to the plasma membrane was determined as the ratio of the mean (IntDen/area) cell surface Egfr fluorescence intensity (Alexa Fluor 647 signal) to total Egfr fluorescence intensity (Emerald GFP signal)^20^. Analysis was performed for ISCs touching multiple Paneth cells (ISC^MP^), which was the case at the crypt bottom, ISCs touching a single Paneth cell at the crypt edge (ISC^SP^), and TA cells with no Paneth cell contact in the TA zone. Paneth cells were identified based on the flattened nucleus shape and on their apical granulation visible in the bright-field image. The mean transport efficiency was calculated from individual organoid cells for each condition. Per mouse and condition, a total of *n* = 10 organoids with an average of 120 crypt cell types per organoid were analyzed.

### Live-cell Egfr transport quantification

Intestinal organoids were grown from Egfr-Em mice on a 14 mm glass-bottom MatTek dish in a Matrigel drop covered by 200 µl ENR medium. New Egfr-Em synthesis was induced as described for the Egfr transport assay. The change into Egf-free media (NR) after ENR^HighE^ pulse was performed on the Nikon CrEST X-Light V3 spinning disk confocal while the organoid was live imaged with full microscope temperature incubation and CO_2_ control. Upon Egfr-Em release, timelapse images were taken every 2 min for a total length of 60 min and vesicle movement was followed during this time.

The timelapse images were manually annotated for individual ISCs through the entire imaging series. Subsequently, each of the ROIs was analyzed to identify the positive Egfr-Em signal and its distribution over time. Briefly, every cell was rotated to the same orientation, and a bisecting line was drawn through the center of the cells. Signal coordinates were normalized to the lateral (fractional distance from the bisecting line to cell sides) and longitudinal (fractional distance from the central line to cell ends) coordinates (see notebooks under “code availability”). For all images, intensity values > 2 were considered a positive signal. For lumen-side analyses, only signal coordinates in the lumen-facing half of the cell were included.

 A two-sided Mann-Whitney U test with Benjamini–Hochberg false discovery rate (FDR) correction to account for multiple testing was used to compare lateral signal distribution between cell types.

### Microtubule assay

CRISPR-depleted Akap9 and, as a control, scramble-targeted organoids were cultured for 5 days in standard ENR medium. Organoids were extracted from the Matrigel by pipetting in ice-cold PBS and pelleted by centrifugation at 300 x *g* for 30 s. At this point, organoid lysates were taken in parallel, and Akap9 RNA was confirmed by RT-qPCR to be stably depleted (see “RT-qPCR” method). In order to investigate microtubule nucleation, organoids were incubated at 37 °C for 1 h with 10 µM nocodazole, which depolymerizes microtubules, followed by a 2-h washout with standard ENR organoid culture medium to re-nucleate microtubules. Organoids were pelleted and PFA fixed after the 1-h nocodazole treatment directly or after 1-h nocodazole treatment and 2 h washout with standard culture medium. Alternatively, Matrigel-extracted organoids were treated for 2 h with 10 µM tubacin (BML-GR362; Enzo Life Sciences) to promote microtubule acetylation via HDAC6 inhibition. Fixation and immunostaining were performed as described in the “immunofluorescence staining” methods part for DAPI (Nucleus), p115 (Golgi), and acetylated tubulin, and quantified as described in the “Confocal microscopy and image analysis” methods part.

### Egfr transport rescue experiment

Intestinal organoids grown from Egfr-Em mice were cultured for 3 days in standard ENR medium. On day 3, organoids were transfected with siRNA targeting Akap9 or with non-targeting scramble siRNA (see “siRNA-mediated gene knockdown” method). Akap9 mRNA knockdown was subsequently validated by RT-qPCR 48 h later. On day 4 the Egfr transport assay was started (see “Egfr transport assay” method) with a 24-h incubation with NR medium without supplemented Egf. On day 5, organoids were then induced for new Egfr synthesis by the long-term incubation with 200 ng/ml Egf (ENR^HighE^) for 6 h. After 4 h, tubacin (BML-GR362; Enzo Life Sciences) was added at a concentration of 10 µM for 2 h. This was followed by the assessment of the Egfr transport efficiency by “releasing” Egfr as described (see “Egfr transport assay” method).

### Lgr5 transport assay

Intestinal organoids were cultured in ENR media in 48-well culture dishes for 4 days. This was followed by 24 h incubation with EN medium without supplemented R-Spondin. Organoids were then induced for Lgr5 synthesis by incubation with 1000 ng/ml R-spondin-1 (ENR^HighR^) for 12 h (Supplementary Fig. 2l), referred to as “pulse”, if not otherwise indicated in the figures. 20 min before the end of the 12 h period, an additional pulse of 2000 ng/ml R-spondin-1 was induced to ensure the depletion of remaining Lgr5 at the surface. Thereafter, transport of newly synthesized Lgr5, referred to as “release”, was induced by an R-Spondin-1 washout step. For this, organoids were scraped from the culture dish and collected into 1.5 ml low-binding microcentrifuge tubes. The organoids were extracted from the Matrigel by pipetting in ice-cold PBS followed by organoid pelleting through centrifugation at 300 x g for 30 s. Advanced DMEM/F12 medium was added, supplemented with 100 µg/ml cycloheximide (Merck) to stop new protein synthesis, and organoids either fixed (0 min release time point) or incubated for the indicated release times at 37 °C.

After 60 min release time, organoids were pelleted and fixed with 4% PFA for 15 min at room temperature (RT). Following fixation, organoids were blocked for 30 min at RT with 5% Normal Goat Serum (Thermo Fisher Scientific) in DPBS buffer (Dulbecco’s PBS 0.01 M + 0.2% BSA) (blocking buffer). The organoids were then incubated in the blocking buffer containing mouse anti-Lgr5 extracellular domain (Thermo Fisher Scientific, MA5-25644, dilution 1:100) overnight. The next day, organoids were washed 3 times in DPBS and incubated for 2 h with Alexa Fluor 647-conjugated anti-mouse secondary antibody (Thermo Fisher Scientific, A-21235, 1:1000) in blocking buffer at RT, followed again by 3x DPBS washes. Thereafter, the organoids were permeabilized using 0.5% Triton X (Sigma) diluted in DPBS buffer and, afterwards, for another 30 min blocked with 5% Normal Goat Serum (Thermo Fisher Scientific) and 0.25% Triton X in DPBS (blocking buffer). Subsequently, organoids were incubated at 4 °C overnight in the blocking buffer containing rabbit anti-Lgr5 intracellular domain (Thermo Fisher Scientific, PA523000, dilution 1:250). The next day, organoids were washed 3 times in DPBS and incubated for 2 h with Alexa Fluor 488-conjugated anti-rabbit secondary antibody (Thermo Fisher Scientific, A-11008, 1:1000) in blocking buffer at RT, followed again by 3x DPBS washes. Thereafter, DAPI (Life Technologies, 1 μg/ml) staining was performed for 30 min at RT. The organoids were taken up in Immu-Mount media (Thermo Fisher Scientific) and placed on a 14 mm glass-bottom MatTek dish with a coverslip on top. Coverslips were sealed with nail polish.

Images of the nucleus (DAPI), total Lgr5 (Alexa Fluor 488), and plasma membrane Lgr5 (Alexa Fluor 647) were acquired by confocal microscopy and analyzed as described for the Egfr transport assay.

### Transferrin assay

The Egfr transport assay consisted of an Egf pulse leading to rapid Egfr internalization into endosomes, from where the receptor is either recycled back to the cell surface or degraded, triggering new Egfr synthesis^20,81^. Transport of proteins from recycling endosomes to the surface takes 2-5 min^87–89^, whereas Golgi-dependent arrival of newly synthesized Egfr occurs at 45 min^20^, suggesting that the 5 min peak of PM-Egfr in old organoids may represent Egfr recycling from endosomes. We combined our Egfr transport assay with a stimulation with Apo-transferrin, which recycles back to the cell surface after internalization via endosomes that are visible as distinct puncta^44^.

Intestinal organoids were cultured as described for the “Egfr transport assay”. Organoids were extracted from the Matrigel using ice-cold PBS and pelleted by centrifugation at 300 x g for 30 s. Alexa Fluor 647 Apo-transferrin Conjugate (Thermo Scientific) was added at a concentration of 25 µg/ml. To investigate Egfr-Em and Apo-transferrin colocalization, organoids were incubated for a total of 15 min with Apo-transferrin-A647 combined with the indicated Egfr-Em release time points. Organoids were thereafter fixed with 4% PFA for 30 min at RT followed by Egfr assay staining (see “Egfr transport assay” method).

### Single-cell sorting and analysis

To isolate single cells, intestinal crypts were dissociated in TrypLE Express (Gibco) with 1000 U/ml of DNase I (Roche) at 32 °C for 90 s. Cells were washed with ice-cold Advanced DMEM/F12, and pelleted by centrifugation for 5 min at 300 x *g*, followed by antibody incubation with: CD45–PE (eBioscience, 12-0451-82, 30-F11), CD31–PE (Biolegend, 102507, Mec13.3), Ter119–PE (Biolegend, 116207, Ter119), EpCAM–APC (eBioscience, 17-5791-82, G8.8) and CD24–Pacific Blue (Biolegend, 101819, M1/69), all diluted 1:250. Finally, unbound antibody was washed away, and cells were suspended in SMEM medium (Sigma) supplemented with 7-AAD (Life Technologies) (2 μg/ml) for live-cell separation and filtered through a 40-μm nylon mesh. Cells were sorted using a FACSAria II or FACSAria Fusion (BD Biosciences). ISCs were gated as live eGFP^high^ and TA cells as Lgr5–eGFP^med/low^; Epcam^+^; CD24^med^(or ^−^); CD31 − ; Ter119^−^; CD45^−^; 7-AAD^−^ and Paneth cells as live CD24^high^; SideScatter^high^; Lgr5–eGFP^−^; Epcam^+^; CD31^−^; Ter119^−^; CD45^−^;7-AAD^−^. The FlowJo software was used for cell population analysis.

### CRISPR–Cas9 gene editing of intestinal organoids

Guide RNAs for the target-gene knockout were designed with the CRISPR design tool (https://chopchop.cbu.uib.no). Akap9 CRISPR guides were designed to introduce a stop codon in exon 2. Guides were cloned into the lentiCRISPR v2 vector. Lentiviral vectors were produced in 293FT cells (Thermo Fisher, R70007) and concentrated with Lenti-X concentrator (Clontech). The 293FT cell line was not authenticated in the laboratory, but it tested negative for mycoplasma. Cultured intestinal organoids were exposed to 3 μM Chir99021 (Sigma) and 1 mM Valproic Acid (Cayman Chemicals). Organoids were mechanically dissociated into small fragments by pipetting, and dissociated into single cells by TrypLE Express treatment supplemented with 1000 U/ml DNase I for 5 min at 37 °C. Cells were washed with cold ADMEM/F12 medium and pelleted by centrifugation at 300 x *g* for 5 min. Cells were then resuspended in transduction medium consisting of ENR medium supplemented with 8 μg/ml polybrene (Sigma-Aldrich), 10 mM nicotinamide (Sigma-Aldrich), 10 μM Y-27632, and 1000 ng/ml R-spondin-1. Samples were mixed with concentrated virus and spinoculated at 600 x *g* at 32 °C for 1 h, followed by 3 h incubation at 37 °C, after which cells were collected and plated in Matrigel overlaid with transduction medium without polybrene. Two days after transduction, 2 μg/ml of puromycin (Sigma-Aldrich) was added every two days to the medium, in order to select the infected clones. Four to five days after starting selection, surviving clones were expanded in normal ENR medium, single clones were picked and expanded, and clonogenic growth was assessed. In experiments comparing gene-edited organoids from young and old mice, organoids were cultured for a maximum of five days after crypt extraction before transduction and experiments were performed as soon as the clones survived. LentiCRISPR v2 was a gift from F. Zhang (Addgene plasmid 52961)^90^. Gene editing was confirmed by sequencing around the target region and by RT-qPCR of the clonogenic organoid culture. Oligonucleotides used for generation of gRNAs: Akap9 (1) CACCGGCCGGGAATCCTGATTGCTC, AAACGAGCAATCAGGATTCCCGGCC; Akap9 (2) CACCGCCATTAAACAGCGAGACGGC, AAACGCCGTCTCGCTGTTTAATGGC; Scramble (1) CACCGCTAAAACTGCGGATACAATC, AAACGATTGTATCCGCAGTTTTAGC; Scramble (2) CACCGAAAACTGCGGATACAATCAG, AAACCTGATTGTATCCGCAGTTTTC.

### siRNA-mediated gene knockdown

Intestinal organoids were cultured for 5 days in standard ENR medium. Organoids were extracted from the Matrigel by pipetting in ice-cold PBS and pelleted by centrifugation at 300 x g for 30 s. Once Matrigel was removed, organoids were transfected with the following siRNAs (Dharmacon) at a final concentration of 25 nM: ON-TARGETplus Non-targeting Negative siRNA- Target Sequence: UGGUUUACAUGUUGUGUGA; ON-TARGETplus Akap9 siRNA- Target Sequence: GCAAAGAGUUAGGCGAAUA. For transfection, organoids were incubated with RNAiMAX (Invitrogen) in DMEM (Gibco) containing 10% normal/dialyzed FBS (Gibco) at 37 °C according to the manufacturer’s ratio. Transfection was performed for 2-4 h, after that organoids were washed once and replated back into Matrigel, followed by 48 h growth in standard ENR conditions. Gene knockdown was assessed by RT-qPCR after 48 h. For this, organoids were extracted from the Matrigel by pipetting in ice-cold PBS and pelleted by centrifugation at 300 x g for 30 seconds, followed by lysis for RNA extraction (see “RT-qPCR” method).

### RT-qPCR

RNA from cultured intestinal organoids or sorted single cells was isolated by LB1 buffer lysis and purified according to the NucleoSpin RNA Plus XS kit manufacturer’s instructions (Macherey-Nagel). Isolated RNA was transcribed to cDNA with the Maxima First Strand cDNA Synthesis Kit according to the manufacturer’s instructions (Thermo Scientific). Gene expression was analyzed by quantitative real-time PCR (RT-qPCR) using iTaq Universal SYBR Green Supermix (Bio-Rad). Samples were run as technical duplicates or triplicates, and fold changes (FC) were calculated relative to Actb or B2m mRNA using the 2-(ΔΔCT) method^91^. The following primers (Sigma) were used:

Actb Forward CCTCTATGCCAACACAGTGC, Actb Reverse CCTGCTTGCTGATCCACATC; Akap9 Forward GTGAGCTCTGCTCGGAAAGT, Akap9 Reverse CTCCGAGCTACATTCATCTGC; B2m Forward ACCGTCTACTGGGATCGAGA, B2m Reverse TGCTATTTCTTTCTGCGTGCAT; Lgr5 Forward CAGTGTTGTGCATTTGGGGG, Lgr5 Reverse CAAGGTCCCGCTCATCTTGA; Chga Forward CAGCAGCTCGTCCACTCTTT, Chga Reverse GACGCACTTCATCACCTTGG; Alpi Forward CAGAACCTGGTGCAAACGTG, Alpi Reverse GTTGGCTCAAAGAGGCCCAT; Dclk1 Forward AGGAGTTTCTGTAATAGCAACCA, Dclk1 Reverse CCGAGTTCAATTCCGGTGGA; Muc2 Forward CAAGTGATTGTGTTTCAGGCTC, Muc2 Reverse TGGAGATGTTCTTGGTGCAG; Lyz1 Forward CTGACTGGGTGTGTTTAGCTCAG, Lyz1 Reverse AATTGATCCCACAGGCATTCTT; Wnt3a Forward TGGAACTGTACCACCATAGATGAC, Wnt3a Reverse ACACCAGCCGAGGCGATG; Fzd7 Forward TGGGTCATTCTGTCCCTCAC, Fzd7 Reverse CGCGGCCAGATGAAAGTA; Egfr Forward CGCCAACTGTACCTATGGATGT, Egfr Reverse GGGCCACCACCACTATGAAG; Egf Forward AGGATCCTGACCCCGAACTT, Egf Reverse ACAGCCGTGATTCTGAGTGG; Tgfa Forward CAAACACTGTGAGTGGTGCC, Tgfa Reverse GGGATCTTCAGACCACTGTCTC; Areg Forward CAGTGCACCTTTGGAAACGA, Areg Reverse ATGTCATTTCCGGTGTGGCT; Ereg Forward TGCTTTGTCTAGGTTCCCACC, Ereg Reverse CGGGGATCGTCTTCCATCTG; Lrig1 Forward: TTGAGGACTTGACGAATCTGC, Lrig1 Reverse: CTTGTTGTGCTGCAAAAAGAGAG.

### RNA sequencing data analysis

RNA sequencing data of FACS-sorted ISCs (Lgr5^high^), TA cells (Lgr5^low^), and Paneth cells were collected and processed in Pentinmikko et al. 2019 and 2022^15,16^. Differential gene expression was analyzed by comparing the mean gene reads between Lgr5^high^, Lgr5^low^, and Paneth cells for key stemness ligands and receptors, Egf pathway receptors and ligands, as well as for secretory pathway genes. Secretory pathway machinery genes for the coat complexes I and II (COPI, COPII) and the Golgi complex were collected from the literature and based on Gene Ontology annotations.

### Immunoblotting

Mouse intestinal-isolated and FACS-sorted single cells were lysed in RIPA buffer with 1x Halt Protease Inhibitor Cocktail (Thermo Fisher Scientific) and 1x PhosSTOP phosphatase inhibitor (Roche). Cells were then sonicated, 5 min centrifugated at 10600 x *g*, and the cleared lysate was used for gel loading. Loading the same number of sorted cells (50,000-100,000 cells) ensured equal protein loading. Precision Plus Protein Standard (Bio-Rad) was used as a protein standard. Samples were run on 4–12% Bis-Tris gels (Life Technologies) and blotted on nitrocellulose membranes. Membranes were then incubated overnight at 4 °C with the following primary antibodies: rabbit polyclonal Erk1/2 (CST, 9102S, 1:1000), rabbit polyclonal Phospho-p44/p42 MAPK 9101S (CST, 9101S, 1:1000), and mouse monoclonal Vinculin (Sigma, V9131, 1:1000). HRP-conjugated anti-rabbit (Sigma-Aldrich, A0545, 1:5000) or anti-mouse (CST, 7076, 1:1000) antibodies were used as secondary antibodies for 1 h at RT. Signal was detected using the Pierce ECL Plus reagent (Thermo Scientific) and visualized on the Bio-Rad luminescence detector. Densitometric analysis was performed using the software Image Lab.

### Immunohistochemistry

Swiss rolls of murine small-intestinal tissues were fixed in 4% buffered formalin, paraffin embedded, and sectioned (5 µm thick). Antigen retrieval was performed by boiling in a pressure cooker using citric acid buffer 0.01 M pH 6.5, followed by blocking with 5% goat serum containing 0.2% Triton X-100. Primary antibody incubation was performed overnight with: rabbit polyclonal Calnexin (Enzo, ADI-SPA-860-D, 1:50), rabbit polyclonal p115 (Proteintech, 13509-1-AP, 1:500), mouse monoclonal E-cadherin (BD, 610181, Clone 36/E, 1:500). After washing, sections were incubated with Alexa Fluor 488-, Alexa Fluor 568-, and Alexa Fluor 647-conjugated anti-mouse or anti-rabbit secondary antibodies (Thermo Fisher Scientific, A-11001, A-11004, A-21235, A-11008, A-11011, A-21245, all diluted 1:1000) at RT for 1 h. Nuclei were stained with DAPI (Life Technologies, 1 μg/ml) for 10 min at RT, and the sections were mounted using ProLong Gold. For hematoxylin staining, paraffin-embedded sections were stained for hematoxylin and eosin and imaged.

### Immunofluorescence staining

For immunofluorescence staining, crypts were isolated, or organoids were grown on glass-bottom dishes (MatTek Corporation) and collected into 1.5 ml low-binding microcentrifuge tubes. The organoids were extracted from the Matrigel by pipetting in ice-cold PBS and pelleted by centrifugation at 300 x *g* for 30 s. Organoids, or from tissue-extracted crypts, were then fixed with 4% PFA for 30 min at RT. For Par3 staining, crypts were fixed with methanol for 15 min on ice. PFA fixed organoids were permeabilized using 0.5% Triton X (Sigma) (0.2% for crypts) diluted in DPBS buffer (Dulbecco’s PBS 0.01 M + 0.2% BSA). PFA/methanol fixed organoids were then blocked for 30 min with 5% Normal Goat Serum (Thermo Fisher Scientific) and 0.25% (0.2% for crypts) Triton X in DPBS (blocking buffer). Subsequently, organoids/crypts were incubated at 4 °C overnight in the blocking buffer containing primary antibodies. The following primary antibodies were used: monoclonal rabbit Olfm4 (Cell Signaling, D6Y5A, dilution 1:100), polyclonal rabbit p115 (Proteintech, 13509-1-AP, 1:500), monoclonal mouse acetylated tubulin (Sigma, T7451, clone 6-11B-1, 1:1000), polyclonal rabbit alpha tubulin (Abcam, ab52866, 1:100), polyclonal rabbit Akap9 (Novus Biologicals, NBP1-89166, 1:100), monoclonal mouse E-cadherin (BD, 610182, Clone 36/E, 1:500), polyclonal chicken GFP (Abcam, ab13970, 1:500), polyclonal rabbit Par3 (Novus Biologicals, NBP1-88861, 1:250), monoclonal mouse ZO-1 (Thermo Fisher Scientific, 33-9100, Clone ZO1-1A12, 1:500). The following day, organoids/crypts were washed 3 times in DPBS and incubated for 2 h at RT with Alexa Fluor 488-, Alexa Fluor 568-, and Alexa Fluor 647-conjugated anti-chicken (A-11039), anti-mouse (A-11001, A-11004, A-21235) or anti-rabbit (A-11008, A-11011, A-21245) secondary antibodies (Thermo Fisher Scientific, all 1:1000) diluted in blocking buffer at RT, followed again by three DPBS washes. Phalloidin-Atto425 (Sigma, 1:500) and DAPI (Life Technologies, 1 μg/ml) staining was performed for 30 min at RT. Golgi NBD C_6_-ceramide staining was performed as described by the manufacturer (Thermo Fisher Scientific). The organoids/crypts were taken up in Immu-Mount media (Thermo Fisher Scientific) and placed on a 14 mm glass-bottom MatTek dish with a coverslip on top. Coverslips were sealed with nail polish.

### Confocal microscopy and image analysis

Confocal microscopy experiments were performed with fixed and immunostained organoid, crypt, or tissue section samples on a Zeiss LSM980-Airy2 confocal microscope, with a GaAsP-PMT detector, Plan-Apochromat 63×1.4Oil and C-Apochromat 40×1.2 W objectives, and the excitation laser lines of 405, 445, 488, 561, 594, 639 nm, using the software ZEN version 3.3. Golgi complex stacks were imaged by Airy-scanning.

Analysis of the confocal images was performed using ImageJ. To analyze the intensity of Egfr-Em within Apo-transferrin puncta structures (Supplementary Fig. 3m), Apo-transferrin structures were determined by thresholding, and Egfr-Em intensity (RawIntDen) inside the detected structures was measured. Only the peripheral structures were considered, where the space between the puncta allowed the segmentation of individual structures.

To quantify p115 intensity distribution within crypt cells (Supplementary Fig. 1c), z-stacks covering the entire thickness of crypts were projected (maximum intensity), and cells were segmented using E-cadherin staining. Cells were then divided into 10 ×10 grids, and p115 intensity per grid was measured.

To quantify Akap9 intensity in organoid crypt cells (Fig. 2b), cells were segmented based on the bright-field image. Mean Akap9 fluorescence intensity (IntDen/area) was measured in ISC^MP^, ISC^SP^ and PCs. ISC^MP^ were identified as being located at the crypt base and as being intercalated between PCs with apical granules and flattened nuclei visible in the bright-field image. ISC^SP^ were identified as being located at the crypt edge and facing only to one side a PC.

To quantify alpha and acetylated tubulin distribution along the organoid crypt cells (Fig. 2c), a line was drawn from the center of the basal plasma membrane side to the center of the apical membrane, and protein intensity was measured as a profile of the grey values (y- axis) and the distance (x-axis).

To quantify the level of acetylated tubulin at Golgi complex stacks of ISCs at the crypt bottom (Figs. 2h, i and Supplementary Fig. 3d, e), the Golgi complex area was segmented based on the p115 staining, and the mean acetylated tubulin fluorescence intensity (IntDen/area) was measured in ISCs. ISCs were identified as being located at the crypt base and as being intercalated between PCs with a flattened nucleus and with apical granules visible in the bright-field image.

### Statistical analysis

For the analysis of in vitro organoid starting efficiency and organoid growth, investigators were blinded whenever possible. Microsoft Excel v.16.16.8 and GraphPad Prism v.8.0.0 were used for statistical analysis and for data visualization. Data groups were compared using a two-tailed unpaired Student’s t-test unless otherwise mentioned. Paired t-tests are indicated in the figure legends and were used to compare treatments between independent biological replicates when the days of the experiment varied (samples of the same day were paired). Resulting P-values are represented in the corresponding figure panels, with *P*-values < 0.05 being considered as significant. For the quantification of acetylated tubulin in crypt-bottom ISCs, statistical outliers were detected using the robust regression and outlier removal (ROUT) method with a false discovery rate of 1% (Q = 1%) and excluded from subsequent analyses.

### Reporting summary

Further information on research design is available in the Nature Portfolio Reporting Summary linked to this article.

## Supplementary information

Supplementary Information 1

Supplementary Data 1

Supplementary Movie 1

Supplementary Movie 2

Reporting Summary

Transparent Peer Review file

## Source data

Source Data File

## Data Availability

All other data are available in the main text or the supplementary materials. Volume EM data will be deposited in EMPIAR, the Electron Microscopy Public Image Archive [https://www.ebi.ac.uk/empiar/]. Correspondence and requests for materials should be addressed to S.S. Source data are provided with this paper.

## Code availability

Vesicle movement analysis notebooks as part of the live-cell Egfr transport quantification are deposited in GitHub [https://github.com/kasperlab/Scharaw_et_al_2024]. To streamline the Golgi orientation quantification analysis, we developed a plugin for MIB that is capable of processing multiple objects and generating results in Excel or MATLAB formats. This plugin is available starting from MIB version 2.93, with detailed instructions and demo datasets accessible at 10.5281/zenodo.21068508.
