# Supplementary material for: Golgi organization regulates stem cell function in the small intestine
Source: Nat Commun. 2026 Jul 29;17:7606. doi: 10.1038/s41467-026-75679-1 (PMC13424321; doi:10.1038/s41467-026-75679-1)
Supplement: Supplementary file 1 — Supplementary Information 1 [file 41467_2026_75679_MOESM1_ESM.pdf]

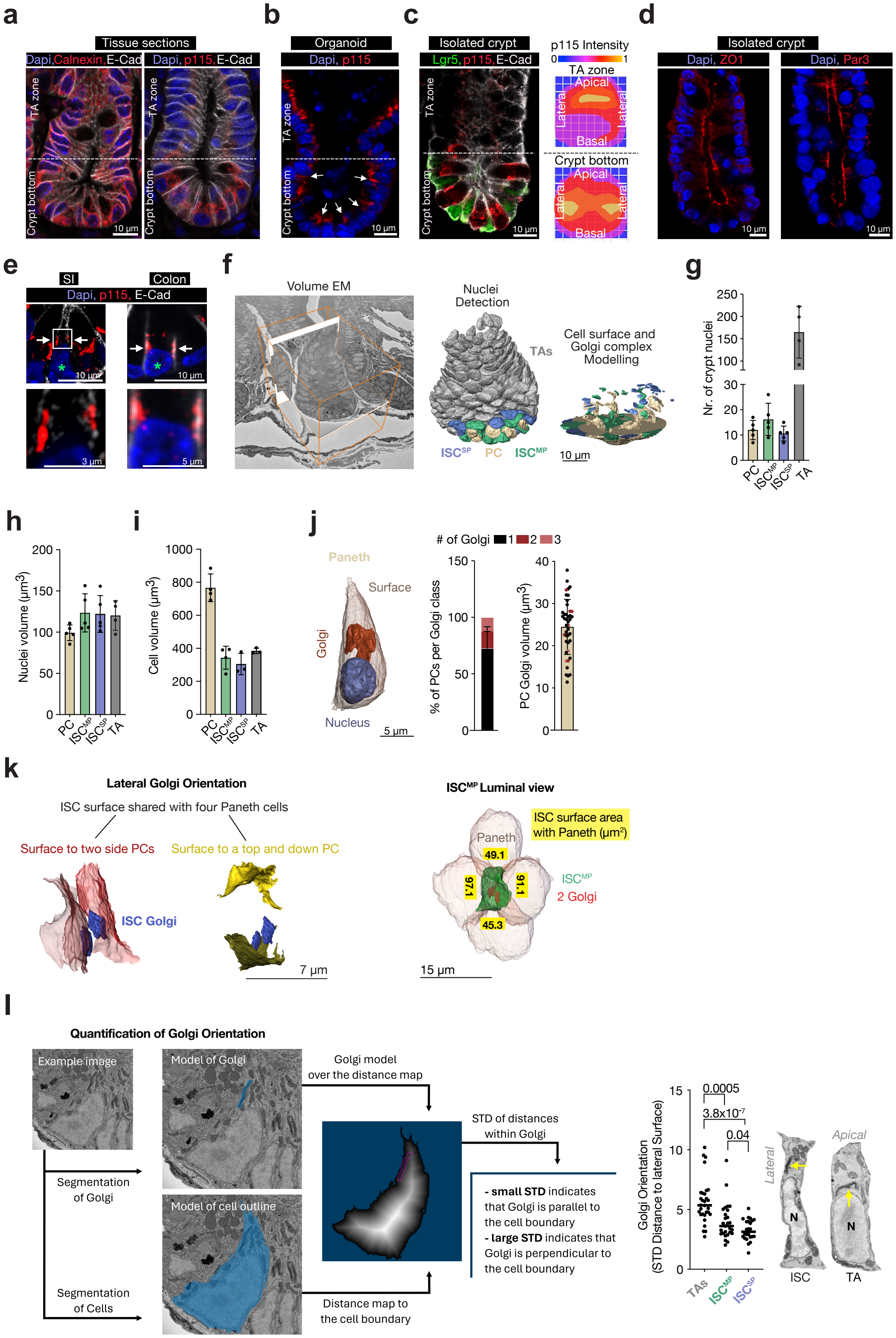

a
Tissue sections
Dapi,Calnexin,E-Cad
Dapi, p115,E-Cad
TA zone
Crypt bottom
10 µm
b
Organoid
Dapi, p115
c
Isolated crypt
Lgr5, p115, E-Cad
p115 Intensity
TA zone
Apical
Lateral
Basal
Crypt bottom
d
Isolated crypt
Dapi, ZO1
Dapi, Par3
e
SI
Colon
Dapi, p115, E-Cad
3 µm
5 µm
f
Volume EM
Nuclei Detection
TAs
Cell surface and Golgi complex Modelling
ISC^SP PC ISC^MP
g
Nr. of crypt nuclei
PC ISC^MP ISC^SP TA
h
Nuclei volume (µm3)
i
Cell volume (µm3)
j
Paneth
Surface
Golgi
Nucleus
# of Golgi 1 2 3
% of PCs per Golgi class
PC Golgi volume (µm3)
k
Lateral Golgi Orientation
ISC surface shared with four Paneth cells
Surface to two side PCs
Surface to a top and down PC
ISC Golgi
7 µm
ISC^MP Luminal view
ISC surface area with Paneth (µm2)
Paneth
49.1
97.1
91.1
45.3
ISC^MP
2 Golgi
15 µm
l
Quantification of Golgi Orientation
Example image
Model of Golgi
Model of cell outline
Segmentation of Golgi
Segmentation of Cells
Golgi model over the distance map
Distance map to the cell boundary
STD of distances within Golgi
- small STD indicates that Golgi is parallel to the cell boundary
- large STD indicates that Golgi is perpendicular to the cell boundary
Golgi Orientation (STD Distance to lateral Surface)
0.0005
3.8x10-7
0.04
TAs ISC^MP ISC^SP
Lateral
Apical
N
ISC
TA

Supplementary Figure 1. Intestinal stem cells orient their Golgi laterally towards Paneth cells. (a, b) Immunostaining of small intestinal (SI) tissue sections and organoids. Endoplasmic reticulum and Golgi were identified using Calnexin and p115 respectively, and the cell surface by E-cadherin (n=3 mice). **(c)** Immunostaining of SI crypts isolated from Lgr5-eGFP-IRES-CreERT2 mice stained for GFP to visualize Lgr5-eGFP cells, p115 (Golgi), and E-cadherin (plasma membrane) (n=3 mice). **(d)** SI crypts stained for Par3/ZO1 as apical markers (n=3 mice). **(e)** Golgi staining of ISCs (green asterisk) in SI and colon crypts (n=3 mice). ISCs were identified by their columnar shape and crypt base localization. **(f)** Volume EM on SI tissue and corresponding models. ISCs contacting multiple Paneth cells (PC) are referred to as ISC^{MP}, those contacting a single Paneth cell as ISC^{SP}, and transient amplifying progenitor cells as TA. **(g)** Volume EM-modeled nuclei counts (n=4 mice), modeled **(h)** nuclei volumes and **(i)** cell volumes. Each dot represents the mean of a single mouse crypt. **(j)** Representative Golgi morphology with Golgi number and Golgi volume of PCs resolved by Volume EM (n=5 mice, each PC Golgi represents a single dot). **(k)** Representative images of the ISC shown in Fig. 1a with split Golgi (blue) sharing two large surface sides (red) and two smaller surface sides (yellow) with the surrounding four Paneth cells. **(l)** Overview and results of the Golgi orientation analysis (see methods) of Volume EM-imaged Golgi complexes in relation to the lateral surface (n=3 mice). Each dot represents a single Golgi orientation. All data are presented as indicated ± s.d. and conditions compared by two-tailed unpaired Student's t-test. $P < 0.05$ is considered significant.

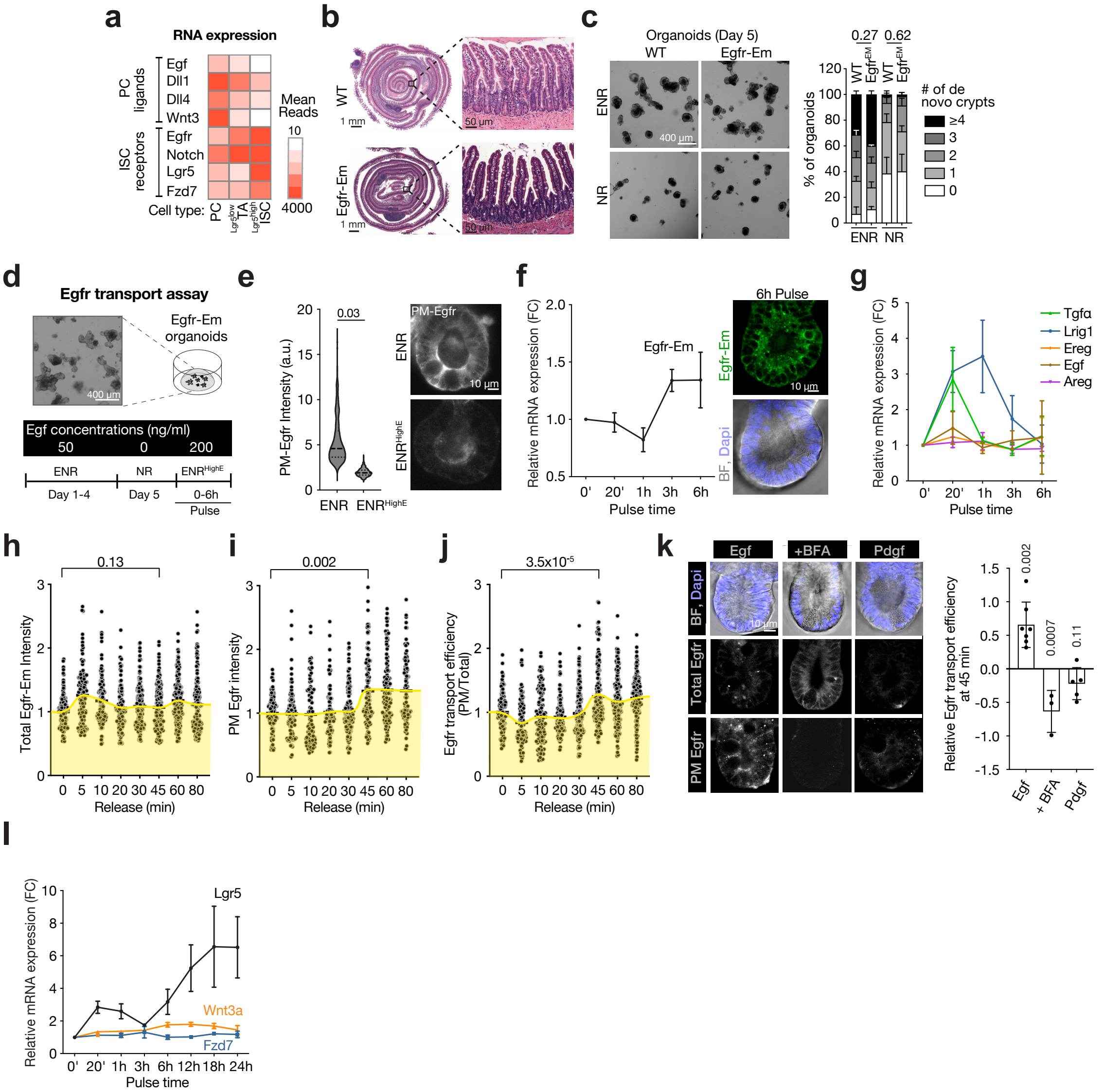
a
RNA expression
PC ligands
Egf
Dll1
Dll4
Wnt3
ISC receptors
Egfr
Notch
Lgr5
Fzd7
Cell type:
PC
TA
ISC
Mean Reads
10
4000
b
WT
1 mm
50 µm
Egfr-Em
c
Organoids (Day 5)
WT
Egfr-Em
ENR
NR
400 µm
0.27
0.62
% of organoids
# of de novo crypts
≥4
3
2
1
0
d
Egfr transport assay
Egfr-Em organoids
400 µm
Egf concentrations (ng/ml)
50
0
200
ENR
NR
Day 1-4
Day 5
0-6h
Pulse
e
0.03
PM-Egfr Intensity (a.u.)
PM-Egfr
ENR
10 µm
f
6h Pulse
Egfr-Em
Relative mRNA expression (FC)
Pulse time
BF, Dapi
10 µm
g
Tgfα
Lrig1
Ereg
Egf
Areg
Relative mRNA expression (FC)
Pulse time
h
0.13
Total Egfr-Em Intensity
Release (min)
i
0.002
PM Egfr intensity
Release (min)
j
3.5x10-5
Egfr transport efficiency (PM/Total)
Release (min)
k
Egf
+BFA
Pdgf
BF, Dapi
Total Egfr
PM Egfr
10 µm
0.002
0.0007
0.11
Relative Egfr transport efficiency at 45 min
Egf
+ BFA
Pdgf
l
Lgr5
Wnt3a
Fzd7
Relative mRNA expression (FC)
Pulse time

Supplementary Figure 2. Establishment of receptor transport assays in intestinal organoids. **(a)** RNA expression of known PC ligands and ISC receptor genes. **(b)** H&E stained Egfr-Em and wild-type (WT) mouse small intestines with zoomed-in ileum region (n=3 mice/group). **(c)** Regenerative growth capacity of Egfr-Em and WT organoids at day 5, grown with (ENR) or without Egf (NR) (n=3 mice/group). **(d)** Strategy to induce *de novo* Egfr-Em synthesis in organoid cells (see methods). **(e)** Plasma membrane (PM) Egfr depletion induced by a 6 hour 200 ng/ml pulse (ENR^{HighE}) (n=3 mice). **(f,g)** RT-qPCR of Egfr-Em organoid lysates treated for the indicated times with ENR^{HighE} (pulse) relative to time point 0 (n=5 mice). **(h-j)** Quantified Egfr intensities. Each dot represents a single cell from 10 organoids/mouse. For statistical comparisons, cell intensities were averaged per mouse and compared (n = 5 biologically independent mice). **(k)** Egfr transport efficiency under ENR^{HighE} pulse with brefeldin A (BFA), or under $ENR^{HighPdgf}$ pulse. Data show the mean cellular efficiency of 10 organoids/mouse (Egf=6 mice, +BFA=3 mice, Pdgf=4 mice). **(l)** RT-qPCR of R-Spondin-1-treated organoids under ENR^{HighR} pulse (n=4 mice). Unless otherwise mentioned, data are presented as mean ± s.d. and conditions compared by two-tailed unpaired Student's t-test. $P < 0.05$ is considered significant.

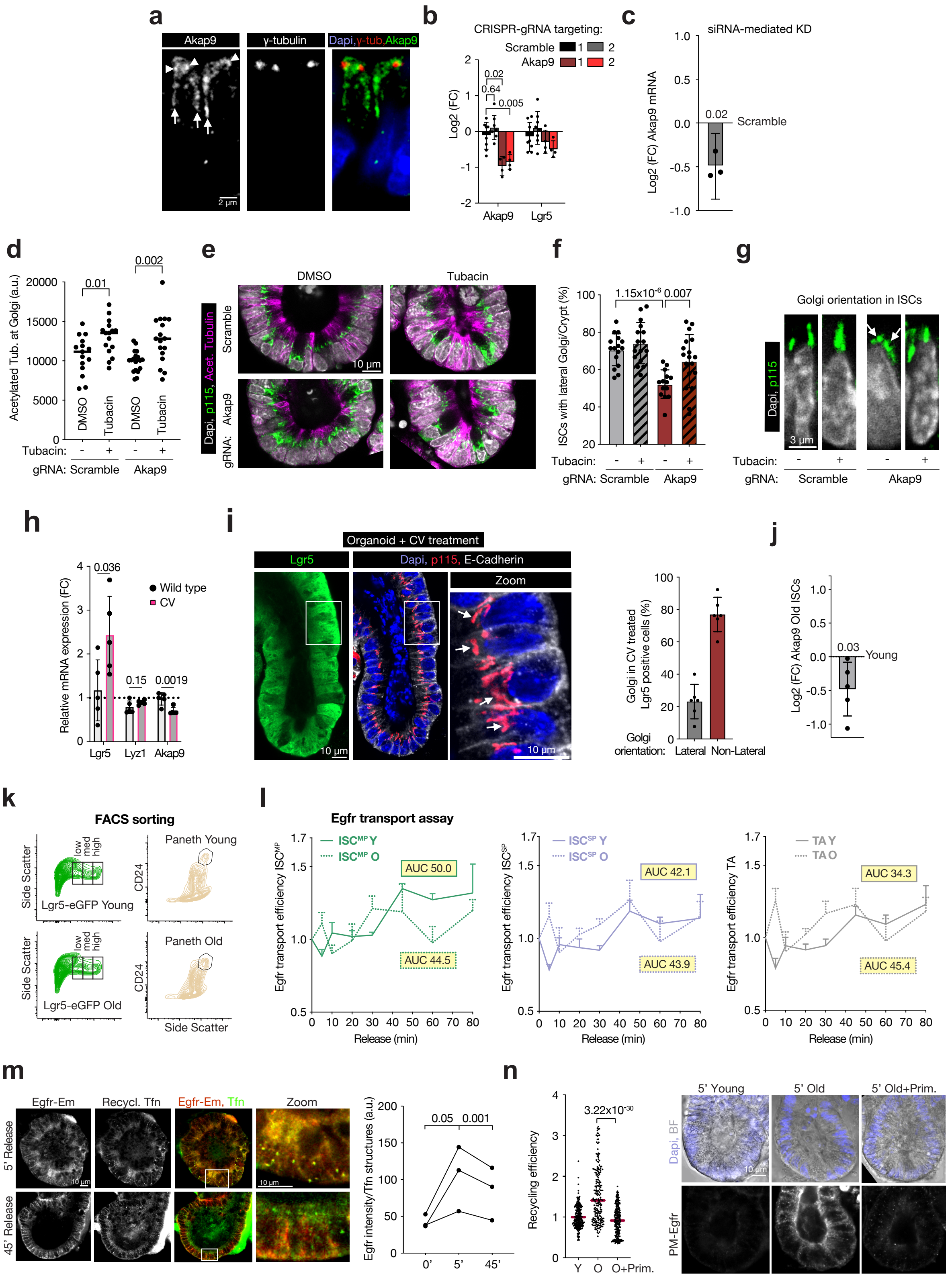
a
Akap9
γ-tubulin
Dapi,γ-tub,Akap9
2 µm
b
CRISPR-gRNA targeting:
Scramble 1 2
Akap9 1 2
Log2 (FC)
0.02
0.64
0.005
Akap9
Lgr5
c
siRNA-mediated KD
Log2 (FC) Akap9 mRNA
0.02
Scramble
d
Acetylated Tub. at Golgi (a.u.)
0.01
0.002
DMSO
Tubacin
Tubacin:
gRNA: Scramble
Akap9
e
DMSO
Tubacin
Dapi, p115, Acet. Tubulin
gRNA: Scramble
Akap9
10 µm
f
ISCs with lateral Golgi/Crypt (%)
$1.15x10^{-6}$
0.007
Tubacin:
gRNA: Scramble
Akap9
g
Golgi orientation in ISCs
Dapi, p115
3 µm
Tubacin:
gRNA:
Scramble
Akap9
h
Relative mRNA expression (FC)
0.036
0.15
0.0019
Wild type
CV
Lgr5
Lyz1
Akap9
i
Organoid + CV treatment
Lgr5
Dapi, p115, E-Cadherin
Zoom
10 µm
Golgi in CV treated Lgr5 positive cells (%)
Golgi orientation:
Lateral
Non-Lateral
j
Log2 (FC) Akap9 Old ISCs
0.03
Young
k
FACS sorting
Side Scatter
low med high
Lgr5-eGFP Young
Lgr5-eGFP Old
Paneth Young
Paneth Old
CD24
Side Scatter
l
Egfr transport assay
Egfr transport efficiency ISC^MP
ISC^MP Y
ISC^MP O
AUC 50.0
AUC 44.5
Egfr transport efficiency ISC^SP
ISC^SP Y
ISC^SP O
AUC 42.1
AUC 43.9
Egfr transport efficiency TA
TA Y
TA O
AUC 34.3
AUC 45.4
Release (min)
m
Egfr-Em
Recycl. Tfn
Egfr-Em, Tfn
Zoom
5' Release
45' Release
10 µm
Egfr intensity/Tfn structures (a.u.)
0.05
0.001
0'
5'
45'
n
Recycling efficiency
$3.22x10^{-30}$
Y
O
O+Prim.
5' Young
5' Old
5' Old+Prim.
Dapi, BF
PM-Egfr

Supplementary Figure 3. Old stem cells display changed Egfr transport dynamics. (a) Immunostaining of Akap9 and gamma-tubulin in organoids demonstrates Akap9 localization pericentriolar (arrowheads) and at extended lateral structures (arrows) (n=3 mice). **(b)** RT-qPCR analysis of Akap9 vs. Scramble-targeted small intestinal organoid lysates. **(c)** RT-qPCR analysis of Akap9 mRNA expression in intestinal organoids transfected with siRNA targeting Akap9 vs. Scramble non-targeting siRNA (organoids from n=3 mice). **(d)** Quantification and **(e)** representative images of acetylated tubulin at p115-positive Golgi complex stacks of crypt bottom ISCs in Akap9 CRISPR-depleted vs. Scr-targeted organoids treated for 2 hours with 10 μM Tubacin vs. DMSO control. Each data point in (d) represents the mean measured intensity of acetylated tubulin at p115-positive Golgi complexes of ISCs per organoid. (n=3 independent experiments with two-tailed unpaired Student's t-test comparing organoids). **(f)** Quantification and **(g)** representative images of the percentage of lateral oriented p115-positive Golgi complex stacks of ISCs per crypt in Akap9 CRISPR-depleted vs. Scr-targeted organoids treated for 2 hours with 10 μM Tubacin vs. DMSO control. Each data point in (f) represents the mean percentage of lateral Golgi stacks of ISCs per organoid. (n=3 independent experiments with two-tailed unpaired Student's t-test comparing organoids). **(h)** RT-qPCR analysis of Akap9, Lgr5 (ISCs) and lysozyme (PCs) mRNA expression in intestinal organoids grown with ENR media supplemented with CHIR99021 and valproic acid (CV) (n=5 experimental replicates). **(i)** Intestinal organoids from Lgr5-eGFP-IRES-CreERT2 mouse crypts grown with ENR media supplemented with CV and immunostained with p115 (Golgi) were analyzed for Golgi orientation. Arrows point to collapsed Golgi complexes scored as non-lateral (organoids from n=3 mice). **(j)** RT-qPCR analysis of Akap9 mRNA expression in FACS-isolated Lgr5+ ISCs from old vs. young mice (n=5 mice). **(k)** FACS gating strategy for ISCs ($Lgr5^{high}$), TAs ($Lgr5^{med}$, $Lgr5^{low}$) and PCs. **(l)** Egfr transport efficiency of old vs. young Egfr-Em mouse organoids in intestinal stem cells with multiple Paneth cell neighbors (ISC^{MP}, left panel), with single Paneth neighbor (ISC^{SP}, middle panel) and in transient amplifying progenitor cells (TA, right panel). Data points represent means ± s.e.m. and displayed is the area under the curve (AUC) (n=4 mice). **(m)** Egfr-Em localization in comparison to recycling transferrin (Tfn) displays a higher intensity of Egfr-Em in Tfn-positive recycling puncta at 5 min than at 45 min post Egfr-release (n=3 mice). **(n)** Increased Egfr-Em recycling in cells of Egfr-Em organoid crypts from old mice can be reverted with the Egfr recycling inhibitor primaquine. Representative images show recycled Egfr staining at the plasma membrane. Data represent single cells (n=3 mice). All data are presented as mean ± s.d. and conditions compared by two-tailed unpaired Student's t-test. P values shown in corresponding panels. $P < 0.05$ is considered significant.

Gene ID	Mean ISC (Lgr5^{high})	Mean TA (Lgr5^{low})	Mean Paneth
Egf	13.69372781	54.29764575	386.6113978
Dll1	548.4330078	1098.584295	1371.326157
Dll4	142.085142	367.0936161	832.4338843
Wnt3	39.43843685	91.15716003	1229.519954
Egfr	1203.930449	891.3235656	546.3687041
Notch1	3979.728761	1641.051683	643.2336797
Lgr5	3584.058658	223.1941131	667.2710225
Fzd7	987.42969	465.6710311	317.7017361

Supplementary Table 1. Mean RNA-sequencing reads of known receptors and their corresponding ligands crucial for ISC function in FACS-sorted Paneth cells (PCs), ISCs (Lgr5^{high}) and TA progenitor (Lgr5^{low}) cells. Among these, epidermal growth factor receptor (Egfr) and Leucine-rich repeat-containing G protein-coupled receptor 5 (Lgr5) show a clear gradual increase in expression from Paneth cells to TAs and ISCs. The color scheme indicates low expression in green and high expression in red.

Gene ID	Mean ISC ($Lgr5^{high}$)	Mean TA ($Lgr5^{low}$)	Mean Paneth
Egf	13.69372781	54.29764575	386.61139775
Areg	2.30090013	2.22921280	27.46980408
Ereg	3.37035710	6.43102838	20.15063022
Tgfa	241.63252535	258.14299466	348.20707939
Epgn	0.00000000	0.00000000	0.00000000
Hbegf	40.13842376	53.23010826	69.81037991
Btc	98.02460589	68.11966489	57.79267301
Nrg1	0.00000000	8.31477310	33.26834067
Nrg2	0.00000000	1.35266404	0.00000000
Nrg3	0.00000000	0.00000000	0.16829375
Nrg4	162.30762118	69.83632861	35.32836811
Lrig1	4189.57673248	2138.15701436	2070.92405038
Lrig3	774.71005667	909.05597787	618.99626937
Egfr	1203.93044942	891.32356556	546.36870406
Erbb2	1824.96543669	1925.61582375	2004.04985917
Erbb3	3045.03781914	2831.61057774	4505.48995536
Erbb4	0.00000000	0.00000000	2.15766869
Cblb	635.94221546	470.81896806	851.82386453
Nedd4	8819.80526369	5722.28149149	5030.61902589
Src	798.33450183	718.11535681	1635.30647920

Supplementary Table 2. Mean RNA-sequencing reads of known Egf pathway receptors and ligands in FACS-sorted Paneth cells, ISCs ($Lgr5^{high}$) and TA progenitor ($Lgr5^{low}$) cells. RNA expression levels demonstrate that Egf receptor enrichment in FACS-sorted ISCs ($Lgr5^{high}$) is specific to Egfr amongst the Egf receptor family members.

Class	mgi_symbol	Mean ISC (Lgr5^{high})	Mean TA (Lgr5^{low})	Mean Paneth
COPI vesicle	**Copa**	2377.472173	3580.063535	6027.532456
COPI vesicle	**Copb1**	1313.050792	1743.235378	2231.849831
COPI vesicle	**Copb2**	1792.935067	2885.19685	4154.865826
COPI vesicle	**Cope**	844.2998237	1217.855698	1757.038097
COPI vesicle	**Copz1**	1082.422576	1386.680378	1732.557574
COPI vesicle	**Copg1**	1462.343808	2096.154863	3133.804156
COPI vesicle	**Copg2**	644.4592736	591.4029234	572.5190744
COPI vesicle	**Arcn1**	2075.570804	2966.807226	5123.524536
COPI vesicle	**Arf1**	2098.574818	3373.920188	5302.263387
COPI vesicle	**Gbf1**	1137.131029	1653.0586	3071.30643
COPII vesicle	**Sec23a**	509.5171072	970.7355747	1641.705707
COPII vesicle	**Sec23b**	609.4877127	1118.558104	1666.160454
COPII vesicle	**Sec24a**	1362.882222	2257.794597	3282.747037
COPII vesicle	**Sec24b**	1141.516139	1089.901186	921.2856138
COPII vesicle	**Sec24c**	1661.369882	2122.099465	2622.848801
COPII vesicle	**Sec24d**	100.7284558	780.915395	3319.307495
COPII vesicle	**Sec31a**	3020.177809	4514.827641	7495.594723
COPII vesicle	**Sec31b**	14.34615541	15.84983372	25.77333226
COPII vesicle	**Preb**	928.7819013	1381.015159	1896.511733
COPII vesicle	**Sec13**	636.1685871	1253.752255	1037.095372
COPII vesicle	**Sec16a**	1624.392943	2274.674955	4553.692257
COPII vesicle	**Sec16b**	391.5419983	502.2853707	1120.086579
COPII vesicle	**Sar1a**	755.2390323	981.6972276	1286.741645
COPII vesicle	**Sar1b**	458.7984067	760.6511534	879.1999535
Golgi complex	**Gorasp1**	348.5070663	339.3824426	563.5124286
Golgi complex	**Gorasp2**	1038.236311	1571.918546	2159.965103
Golgi complex	**Golga2**	621.0196343	778.6003177	1539.631751
Golgi complex	**Uso1**	887.7792938	1327.89096	2672.37623
Golgi complex	**Rabac1**	199.4126229	222.5566211	453.4897883
Golgi complex	**Golga1**	629.2006484	714.4101277	945.1439129
Golgi complex	**Golga5**	424.0760214	491.9190194	820.2203187
Golgi complex	**Blzf1**	229.55786	282.8942441	261.9987871
Golgi complex	**Golga3**	836.0157192	886.9097301	1266.317831
Golgi complex	**Golga4**	3848.891133	4028.284007	6397.239395
Golgi complex	**Golgb1**	2572.105393	2875.751299	5334.186068
Golgi complex	**Cux1**	1648.582401	1508.119638	1408.737331
Golgi complex	**Gcc1**	258.7043863	274.4885037	613.8602494
Golgi complex	**Gcc2**	4.802613413	7.596484184	5843.640987

Golgi complex	**Golga7**	403.8676217	471.0327544	626.8001943
Golgi complex	**Acbd3**	561.4640456	797.9052031	1215.695703
Golgi complex	**Akap9**	5195.545271	4131.72124	4623.253913
Golgi complex	**Trip11**	1601.621952	1899.342724	2783.732914
Golgi complex	**Scoc**	75.7881163	93.43981085	265.8921052
Golgi complex	**Gorab**	139.4360653	156.1936353	175.9775028
Golgi complex	**Tmf1**	804.6493396	1014.813316	1986.727674
Golgi complex	**B3gat3**	230.7501971	221.7867911	390.6131478

Supplementary Table 3. Mean RNA-sequencing reads of known secretory pathway machinery genes, including Golgi, COPI and COPII vesicle carrier genes, in FACS-sorted Paneth cells, ISCs ($Lgr5^{high}$) and TA progenitor ($Lgr5^{low}$) cells.

Class	mgi_symbol	log2 Fold Change ISC (Lgr5^{high}) vs. TA (Lgr5^{low})
COPII vesicle	**Sec24d**	-2.954694951
COPII vesicle	**Sec13**	-0.978771254
COPII vesicle	**Sec23a**	-0.929947777
COPII vesicle	**Sec23b**	-0.875971158
COPII vesicle	**Sar1b**	-0.729374581
COPII vesicle	**Sec24a**	-0.728253351
COPI vesicle	**Copb2**	-0.686346513
COPI vesicle	**Arf1**	-0.685015948
Golgi complex	**Gcc2**	-0.661512179
Golgi complex	**Gorasp2**	-0.598391612
COPI vesicle	**Copa**	-0.590556736
Golgi complex	**Uso1**	-0.580863719
COPII vesicle	**Sec31a**	-0.580037424
COPII vesicle	**Preb**	-0.572317391
COPI vesicle	**Gbf1**	-0.539739365
COPI vesicle	**Cope**	-0.528515882
COPI vesicle	**Copg1**	-0.519462767
COPI vesicle	**Arcn1**	-0.515403041
Golgi complex	**Acbd3**	-0.507023714
COPII vesicle	**Sec16a**	-0.485759738
COPI vesicle	**Copb1**	-0.408844657
COPII vesicle	**Sar1a**	-0.378344813
COPII vesicle	**Sec16b**	-0.359340192
COPI vesicle	**Copz1**	-0.357371458
COPII vesicle	**Sec24c**	-0.353118972
Golgi complex	**Tmf1**	-0.334782247
Golgi complex	**Golga2**	-0.326244051
Golgi complex	**Scoc**	-0.302065705
Golgi complex	**Blzf1**	-0.301404993
Golgi complex	**Trip11**	-0.2459666
Golgi complex	**Golga7**	-0.221944896
Golgi complex	**Golga5**	-0.214097926
Golgi complex	**Golga1**	-0.183232375
Golgi complex	**Gorab**	-0.163731903
Golgi complex	**Golgb1**	-0.160989155
Golgi complex	**Rabac1**	-0.158415686
COPII vesicle	**Sec31b**	-0.143803541
Golgi complex	**Gcc1**	-0.085441211

Golgi complex	**Golga3**	-0.085257205
Golgi complex	**Golga4**	-0.065722537
Golgi complex	**Gorasp1**	0.038275977
Golgi complex	**B3gat3**	0.057158435
COPII vesicle	**Sec24b**	0.066753915
COPI vesicle	**Copg2**	0.123947816
Golgi complex	**Cux1**	0.128475118
Golgi complex	**Akap9**	0.330532246

Supplementary Table 4. Relative RNA expression fold changes between ISCs ($Lgr5^{high}$) vs. TA progenitor ($Lgr5^{low}$) cells of known secretory pathway machinery genes including of Golgi, COPI and COPII vesicle carrier genes.

Class	mgi_symbol	log2 Fold Change ISC (Lgr5^{high}) vs. PC
Golgi complex	**Gcc2**	-10.24883215
COPII vesicles	**Sec24d**	-5.042339077
Golgi complex	**Scoc**	-1.810797387
COPII vesicles	**Sec23a**	-1.687993041
Golgi complex	**Uso1**	-1.589850167
COPII vesicles	**Sec16b**	-1.516371282
COPII vesicles	**Sec16a**	-1.487136131
COPII vesicles	**Sec23b**	-1.4508583
COPI vesicles	**Gbf1**	-1.433453957
COPI vesicles	**Copa**	-1.342139059
COPI vesicles	**Arf1**	-1.337198438
COPII vesicles	**Sec31a**	-1.311409462
Golgi complex	**Golga2**	-1.309874541
Golgi complex	**Tmf1**	-1.303962023
COPI vesicles	**Arcn1**	-1.303628452
COPII vesicles	**Sec24a**	-1.268242691
Golgi complex	**Gcc1**	-1.246605736
COPI vesicles	**Copb2**	-1.212478648
Golgi complex	**Rabac1**	-1.185313329
Golgi complex	**Acbd3**	-1.114516612
COPI vesicles	**Copg1**	-1.099632484
COPI vesicles	**Cope**	-1.057318155
Golgi complex	**Gorasp2**	-1.056873154
Golgi complex	**Golgb1**	-1.052318391
COPII vesicles	**Preb**	-1.029936532
Golgi complex	**Golga5**	-0.951688572
COPII vesicles	**Sar1b**	-0.938330929
COPII vesicles	**Sec31b**	-0.845214913
Golgi complex	**Trip11**	-0.797487144
COPII vesicles	**Sar1a**	-0.768717182
COPI vesicles	**Copb1**	-0.765317235
Golgi complex	**B3gat3**	-0.75940863
Golgi complex	**Golga4**	-0.733006607
COPII vesicles	**Sec13**	-0.70506753
Golgi complex	**Gorasp1**	-0.693259522
COPI vesicles	**Copz1**	-0.67863946
COPII vesicles	**Sec24c**	-0.658761337
Golgi complex	**Golga7**	-0.634123138

Golgi complex	**Golga3**	-0.599037576
Golgi complex	**Golga1**	-0.587013861
Golgi complex	**Gorab**	-0.335787241
Golgi complex	**Blzf1**	-0.190702303
Golgi complex	**Akap9**	0.168366569
COPI vesicles	**Copg2**	0.170765431
Golgi complex	**Cux1**	0.226823363
COPII vesicles	**Sec24b**	0.309230867

Supplementary Table 5. Relative RNA expression fold changes between ISCs (Lgr5^{high}) vs. PC cells of known secretory pathway machinery genes including Golgi, COPI and COPII vesicle carrier genes.

Gene ID	Mean Young ISC (Lgr5^{high})	Mean Young TA (Lgr5^{low})	Mean Old ISC (Lgr5^{high})	Mean Old TA (Lgr5^{low})	Mean Young Paneth	Mean Old Paneth
Akap9	5195.54527	4131.72124	4271.6068	3475.59099	4623.25391	3957.9921

Supplementary Table 6. Mean Akap9 RNA-sequencing reads of FACS-sorted Paneth cells, ISCs (Lgr5^{high}) and TA (Lgr5^{low}) cells isolated from young and old mice.

Gene ID	FC ISC ($Lgr5^{high}$) vs. TA ($Lgr5^{low}$) (Young)	FC ISC ($Lgr5^{high}$) vs. Paneth (Young)	FC ISC ($Lgr5^{high}$) vs. ISC ($Lgr5^{high}$) (Old vs Young)
Akap9	1.2574772	1.1237854	0.82216718

Supplementary Table 7. Akap9 RNA expression fold changes between ISCs ($Lgr5^{high}$) vs. TA progenitors ($Lgr5^{low}$)/PC cells isolated from young mice; and Akap9 RNA expression fold changes in ISCs ($Lgr5^{high}$) isolated from young and old mice.
