# Supplementary material for: Golgi organization regulates stem cell function in the small intestine
Source: Nat Commun. 2026 Jul 29;17:7606. doi: 10.1038/s41467-026-75679-1 (PMC13424321; doi:10.1038/s41467-026-75679-1)
Supplement: Supplementary file 5 — Reporting Summary [file 41467_2026_75679_MOESM5_ESM.pdf]

nature portfolio

Corresponding author(s): Sandra Scharaw and Pekka Katajisto

Last updated by author(s): 17th July 2026

Reporting Summary

Nature Portfolio wishes to improve the reproducibility of the work that we publish. This form provides structure for consistency and transparency in reporting. For further information on Nature Portfolio policies, see our Editorial Policies and the Editorial Policy Checklist.

Statistics

For all statistical analyses, confirm that the following items are present in the figure legend, table legend, main text, or Methods section.

n/a	Confirmed	
☐	☒	The exact sample size (n) for each experimental group/condition, given as a discrete number and unit of measurement
☐	☒	A statement on whether measurements were taken from distinct samples or whether the same sample was measured repeatedly
☐	☒	The statistical test(s) used AND whether they are one- or two-sided<br>*Only common tests should be described solely by name; describe more complex techniques in the Methods section.*
☒	☐	A description of all covariates tested
☐	☒	A description of any assumptions or corrections, such as tests of normality and adjustment for multiple comparisons
☐	☒	A full description of the statistical parameters including central tendency (e.g. means) or other basic estimates (e.g. regression coefficient) AND variation (e.g. standard deviation) or associated estimates of uncertainty (e.g. confidence intervals)
☐	☒	For null hypothesis testing, the test statistic (e.g. F, t, r) with confidence intervals, effect sizes, degrees of freedom and P value noted<br>*Give P values as exact values whenever suitable.*
☒	☐	For Bayesian analysis, information on the choice of priors and Markov chain Monte Carlo settings
☒	☐	For hierarchical and complex designs, identification of the appropriate level for tests and full reporting of outcomes
☒	☐	Estimates of effect sizes (e.g. Cohen's d, Pearson's r), indicating how they were calculated

Our web collection on statistics for biologists contains articles on many of the points above.

Software and code

Policy information about availability of computer code

Data collection	FACSDiva versions 7 and 8<br>ImageJ2 Version 2.0.0-rc-69/1.52p and 2.16.0/1.54p<br>QuPath<br>ZEN version 3.3
Data analysis	Microsoft Excel Version 16.110.2<br>Adobe Illustrator Version 28.5<br>GraphPad Prism 10<br>FACSDiva versions 7 and 8<br>FlowJo v10<br>ImageJ2 Version 2.0.0-rc-69/1.52p and 2.16.0/1.54p<br>ZEN version 3.3.<br>MIB version 2.93<br>Amira version 2025.1<br>Image Lab 6.0

For manuscripts utilizing custom algorithms or software that are central to the research but not yet described in published literature, software must be made available to editors and reviewers. We strongly encourage code deposition in a community repository (e.g. GitHub). See the Nature Portfolio guidelines for submitting code & software for further information.

Data

Policy information about availability of data

All manuscripts must include a data availability statement. This statement should provide the following information, where applicable:

- Accession codes, unique identifiers, or web links for publicly available datasets
- A description of any restrictions on data availability
- For clinical datasets or third party data, please ensure that the statement adheres to our policy

Source data are provided with this paper. All other data are available in the main text or the supplementary materials. Volume EM data will be deposited to EMPIAR, the Electron Microscopy Public Image Archivehttps://www.ebi.ac.uk/empiar/. Correspondence and requests for materials should be addressed to S.S.

Research involving human participants, their data, or biological material

Policy information about studies with human participants or human data. See also policy information about sex, gender (identity/presentation), and sexual orientation and race, ethnicity and racism.

Reporting on sex and gender	*Use the terms sex (biological attribute) and gender (shaped by social and cultural circumstances) carefully in order to avoid confusing both terms. Indicate if findings apply to only one sex or gender; describe whether sex and gender were considered in study design; whether sex and/or gender was determined based on self-reporting or assigned and methods used. Provide in the source data disaggregated sex and gender data, where this information has been collected, and if consent has been obtained for sharing of individual-level data; provide overall numbers in this Reporting Summary. Please state if this information has not been collected. Report sex- and gender-based analyses where performed, justify reasons for lack of sex- and gender-based analysis.*
Reporting on race, ethnicity, or other socially relevant groupings	*Please specify the socially constructed or socially relevant categorization variable(s) used in your manuscript and explain why they were used. Please note that such variables should not be used as proxies for other socially constructed/relevant variables (for example, race or ethnicity should not be used as a proxy for socioeconomic status). Provide clear definitions of the relevant terms used, how they were provided (by the participants/respondents, the researchers, or third parties), and the method(s) used to classify people into the different categories (e.g. self-report, census or administrative data, social media data, etc.) Please provide details about how you controlled for confounding variables in your analyses.*
Population characteristics	*Describe the covariate-relevant population characteristics of the human research participants (e.g. age, genotypic information, past and current diagnosis and treatment categories). If you filled out the behavioural & social sciences study design questions and have nothing to add here, write "See above."*
Recruitment	*Describe how participants were recruited. Outline any potential self-selection bias or other biases that may be present and how these are likely to impact results.*
Ethics oversight	*Identify the organization(s) that approved the study protocol.*

Note that full information on the approval of the study protocol must also be provided in the manuscript.

Field-specific reporting

Please select the one below that is the best fit for your research. If you are not sure, read the appropriate sections before making your selection.

☒ Life sciences ☐ Behavioural & social sciences ☐ Ecological, evolutionary & environmental sciences

For a reference copy of the document with all sections, see nature.com/documents/nr-reporting-summary-flat.pdf

Life sciences study design

All studies must disclose on these points even when the disclosure is negative.

Sample size	Sample size was not predetermined with any statistical test. Sufficient sample sizes were estimated based on previous experiments with the same system and included at a minimum 3 biological independent experiments.
Data exclusions	Statistical outliers were detected in Figure 2h and Supplementary Figure 3d using the robust regression and outlier removal (ROUT) method with a false discovery rate of 1% (Q = 1%) and excluded from subsequent analyses. No other data was excluded for other reasons than for being technically inadequate or unusable due to mistakes.
Replication	All experiments were successfully reproduced with biological replicates or independent technical replicates.
Randomization	Animals were allocated to experimental groups randomly. Organoid cultures were randomly allocated to experimental conditions at the culture well level. Treatments and controls were distributed across plates using randomized layouts to minimize positional and batch effects.
Blinding	Investigators were blinded whenever possible. This was done systematically for all organoid growth quantifications.

Reporting for specific materials, systems and methods

We require information from authors about some types of materials, experimental systems and methods used in many studies. Here, indicate whether each material, system or method listed is relevant to your study. If you are not sure if a list item applies to your research, read the appropriate section before selecting a response.

Materials & experimental systems

n/a	Involved in the study
☐	☒ Antibodies
☐	☒ Eukaryotic cell lines
☒	☐ Palaeontology and archaeology
☐	☒ Animals and other organisms
☒	☐ Clinical data
☒	☐ Dual use research of concern
☒	☐ Plants

Methods

n/a	Involved in the study
☐	☐ ChIP-seq
☐	☒ Flow cytometry
☐	☐ MRI-based neuroimaging

Antibodies

Antibodies used	Primary antibodies used included mouse anti-EGFR extracellular domain (clone 199.12, Thermo Fisher Scientific, MA5-13319, 1:40, IF), mouse anti-LGR5 extracellular domain (Thermo Fisher Scientific, MA5-25644, 1:100, IF), rabbit anti-LGR5 intracellular domain (Thermo Fisher Scientific, PA5-23000, 1:250, IF), mouse anti-CD45-PE (clone 30-F11, eBioscience, 12-0451-82, 1:250, FACS), mouse anti-CD31-PE (clone MEC13.3, BioLegend, 102507, 1:250, FACS), mouse anti-Ter119-PE (clone TER-119, BioLegend, 116207, 1:250, FACS), rat anti-EpCAM-APC (clone G8.8, eBioscience, 17-5791-82, 1:250, FACS), mouse anti-CD24-Pacific Blue (clone M1/69, BioLegend, 101819, 1:250, FACS), rabbit anti-ERK1/2 (Cell Signaling Technology, 9102S, 1:1000, WB), rabbit anti-phospho-ERK1/2 (Cell Signaling Technology, 9101S, 1:1000, WB), mouse anti-vinculin (Sigma, V9131, 1:1000, WB), rabbit anti-calnexin (Enzo Life Sciences, ADI-SPA-860-D, 1:50, IHC), rabbit anti-p115 (Proteintech, 13509-1-AP, 1:500, IF/IHC), mouse anti-E-cadherin (clone 36/E, BD Biosciences, 610181/610182, 1:500, IHC/IF), rabbit anti-OLFM4 (Cell Signaling Technology, D6Y5A, 1:100, IF), mouse anti-acetylated α-tubulin (clone 6-11B-1, Sigma, T7451, 1:1000, IF), rabbit anti-α-tubulin (Abcam, ab52866, 1:100, IF), rabbit anti-AKAP9 (Novus Biologicals, NBP1-89166, 1:100, IF), chicken anti-GFP (Abcam, ab13970, 1:500, IF), rabbit anti-Par3 (Novus Biologicals, NBP1-88861, 1:250, IF), and mouse anti-ZO-1 (clone ZO1-1A12, Thermo Fisher Scientific, 33-9100, 1:500, IF). Secondary antibodies included Alexa Fluor 488, 568, and 647 conjugated anti-mouse, anti-rabbit, anti-chicken IgG (Thermo Fisher Scientific; A-11001, A-11004, A-11008, A-11011, A-21235, A-21245, A-11039; all 1:1000), as well as HRP-conjugated anti-rabbit (Sigma, A0545, 1:5000) and anti-mouse (Cell Signaling Technology, 7076, 1:1000).
Validation	All antibodies used in this study were validated by the respective manufacturers for the reported applications and species, as indicated in the corresponding datasheets and product information. Where available, validation included Western blotting, immunofluorescence, immunohistochemistry, flow cytometry, or immunoprecipitation in mouse or human samples, and was supported by published citations provided by the suppliers. Antibodies were used within the applications and species for which they were validated. In addition, co-staining patterns consistent with known subcellular marker or tissue localization (e.g., EGFR plasma membrane localization, LGR5 expression in intestinal stem cells, p115 Golgi localization, and ZO-1 tight junction localization), single channel stainings and concentration curves were observed under the experimental conditions used, further supporting specificity of antibodies in the present study. No additional antibody validation beyond manufacturer-reported performance and established literature use was performed.

Eukaryotic cell lines

Policy information about cell lines and Sex and Gender in Research

Cell line source(s)	293FT cells were purchased from Thermo Fischer Scientific,R70007, lot# 1745311
Authentication	Cell lines were not authenticated.
Mycoplasma contamination	Cell lines were regularly tested for mycoplasma contamination. All cell lines tested negative.
Commonly misidentified lines (See ICLAC register)	No commonly misidentified cell lines were used (ICLAC Version 8.0).

Animals and other research organisms

Policy information about studies involving animals; ARRIVE guidelines recommended for reporting animal research, and Sex and Gender in Research

Laboratory animals	Lgr5-eGFP-IRES-CreERT2, Egfr-Em and wild-type mice were maintained with a C57BL/6J background and housed at Karolinska Institute and Max Planck Institute of Cell Biology and Genetics in IVC cages at consistent temperature (19-23 °C) and humidity (55 % ± 10 %) under a 12 hours light–dark cycle. Standard chow and water were accessible ad libitum. Aging experiments were performed with animals of 3-6 months (referred as 'young') and of 22 months or older (referred as 'old').

Wild animals	Study did not involve wild animals.
Reporting on sex	Both sexes were used equally in the study.
Field-collected samples	Study did not involve field-collected samples.
Ethics oversight	Animal housing and all experimental procedures were performed in accordance with national and institutional guidelines and regulations. Animal husbandry, breeding and organ extraction used in the project have been approved by the Saxon state authority (Landesdirektion Sachsen, license IDs: DD24-5131/346/3 and 25-5131/554/9 [TVT08/2023]) and are overseen by the Institutional Animal Welfare Officer and the Institutional Animal Welfare Body.

Note that full information on the approval of the study protocol must also be provided in the manuscript.

Plants

Seed stocks	*Report on the source of all seed stocks or other plant material used. If applicable, state the seed stock centre and catalogue number. If plant specimens were collected from the field, describe the collection location, date and sampling procedures.*
Novel plant genotypes	*Describe the methods by which all novel plant genotypes were produced. This includes those generated by transgenic approaches, gene editing, chemical/radiation-based mutagenesis and hybridization. For transgenic lines, describe the transformation method, the number of independent lines analyzed and the generation upon which experiments were performed. For gene-edited lines, describe the editor used, the endogenous sequence targeted for editing, the targeting guide RNA sequence (if applicable) and how the editor was applied.*
Authentication	*Describe any authentication procedures for each seed stock used or novel genotype generated. Describe any experiments used to assess the effect of a mutation and, where applicable, how potential secondary effects (e.g. second site T-DNA insertions, mosiacism, off-target gene editing) were examined.*

ChIP-seq

Data deposition

☐ Confirm that both raw and final processed data have been deposited in a public database such as GEO.

☐ Confirm that you have deposited or provided access to graph files (e.g. BED files) for the called peaks.

Data access links<br>*May remain private before publication.*	*For "Initial submission" or "Revised version" documents, provide reviewer access links. For your "Final submission" document, provide a link to the deposited data.*
Files in database submission	*Provide a list of all files available in the database submission.*
Genome browser session<br>(e.g. UCSC)	*Provide a link to an anonymized genome browser session for "Initial submission" and "Revised version" documents only, to enable peer review. Write "no longer applicable" for "Final submission" documents.*

Methodology

Replicates	*Describe the experimental replicates, specifying number, type and replicate agreement.*
Sequencing depth	*Describe the sequencing depth for each experiment, providing the total number of reads, uniquely mapped reads, length of reads and whether they were paired- or single-end.*
Antibodies	*Describe the antibodies used for the ChIP-seq experiments; as applicable, provide supplier name, catalog number, clone name, and lot number.*
Peak calling parameters	*Specify the command line program and parameters used for read mapping and peak calling, including the ChIP, control and index files used.*
Data quality	*Describe the methods used to ensure data quality in full detail, including how many peaks are at FDR 5% and above 5-fold enrichment.*
Software	*Describe the software used to collect and analyze the ChIP-seq data. For custom code that has been deposited into a community repository, provide accession details.*

Flow Cytometry

Plots

Confirm that:

- [x] The axis labels state the marker and fluorochrome used (e.g. CD4-FITC).
- [x] The axis scales are clearly visible. Include numbers along axes only for bottom left plot of group (a 'group' is an analysis of identical markers).
- [x] All plots are contour plots with outliers or pseudocolor plots.
- [x] A numerical value for number of cells or percentage (with statistics) is provided.

Methodology

Sample preparation: Mouse primary intestinal epithelial cells were isolated by EDTA treatment of minced tissue followed by gentle mechanical dissociation and enzymatic treatment with TrypLE Express enzyme to yield single cell suspension.

Instrument: FACSAriaII and FACSAriaIII Fusion (BD) were used to analyse and collect data.

Software: FACSDiva 7 and 8 were used to collect the data. FlowJo v10 was used for analyzing cellular frequencies.

Cell population abundance: Lgr5+ stem cell populations were ~99 % pure based on fluorescent microscopy of the post-sort fraction. Paneth cells were ~90% pure based on phase contrast microscopy (granulated morphology).

Gating strategy: Single cells were gated by using FSC-A,FSC-W,SSC-A and SSC-W parameters as detailed in the methods. Initially the right population was identified by overlaying Lgr5-EGFP on SSC-A vs FSC-A gate. Similar overlay were used to ensure that doublets were not included in the downstream gating. For fluorescent markers, positive populations were identified by comparing to unstained control sample from the same tissue.

- [x] Tick this box to confirm that a figure exemplifying the gating strategy is provided in the Supplementary Information.

Magnetic resonance imaging

Experimental design

Design type: *Indicate task or resting state; event-related or block design.*

Design specifications: *Specify the number of blocks, trials or experimental units per session and/or subject, and specify the length of each trial or block (if trials are blocked) and interval between trials.*

Behavioral performance measures: *State number and/or type of variables recorded (e.g. correct button press, response time) and what statistics were used to establish that the subjects were performing the task as expected (e.g. mean, range, and/or standard deviation across subjects).*

Acquisition

Imaging type(s): *Specify: functional, structural, diffusion, perfusion.*

Field strength: *Specify in Tesla*

Sequence & imaging parameters: *Specify the pulse sequence type (gradient echo, spin echo, etc.), imaging type (EPI, spiral, etc.), field of view, matrix size, slice thickness, orientation and TE/TR/flip angle.*

Area of acquisition: *State whether a whole brain scan was used OR define the area of acquisition, describing how the region was determined.*

Diffusion MRI ☐ Used ☐ Not used

Preprocessing

Preprocessing software: *Provide detail on software version and revision number and on specific parameters (model/functions, brain extraction, segmentation, smoothing kernel size, etc.).*

Normalization: *If data were normalized/standardized, describe the approach(es): specify linear or non-linear and define image types used for transformation OR indicate that data were not normalized and explain rationale for lack of normalization.*

Normalization template: *Describe the template used for normalization/transformation, specifying subject space or group standardized space (e.g. original Talairach, MNI305, ICBM152) OR indicate that the data were not normalized.*

Noise and artifact removal: *Describe your procedure(s) for artifact and structured noise removal, specifying motion parameters, tissue signals and physiological signals (heart rate, respiration).*

Volume censoring

Define your software and/or method and criteria for volume censoring, and state the extent of such censoring.

Statistical modeling & inference

Model type and settings

Specify type (mass univariate, multivariate, RSA, predictive, etc.) and describe essential details of the model at the first and second levels (e.g. fixed, random or mixed effects; drift or auto-correlation).

Effect(s) tested

Define precise effect in terms of the task or stimulus conditions instead of psychological concepts and indicate whether ANOVA or factorial designs were used.

Specify type of analysis: ☐ Whole brain ☐ ROI-based ☐ Both

Statistic type for inference

(See Eklund et al. 2016)

Specify voxel-wise or cluster-wise and report all relevant parameters for cluster-wise methods.

Correction

Describe the type of correction and how it is obtained for multiple comparisons (e.g. FWE, FDR, permutation or Monte Carlo).

Models & analysis

n/a	Involved in the study
☐	☐ Functional and/or effective connectivity
☐	☐ Graph analysis
☐	☐ Multivariate modeling or predictive analysis

Functional and/or effective connectivity

Report the measures of dependence used and the model details (e.g. Pearson correlation, partial correlation, mutual information).

Graph analysis

Report the dependent variable and connectivity measure, specifying weighted graph or binarized graph, subject- or group-level, and the global and/or node summaries used (e.g. clustering coefficient, efficiency, etc.).

Multivariate modeling and predictive analysis

Specify independent variables, features extraction and dimension reduction, model, training and evaluation metrics.
