# Supplementary material for: Golgi organization regulates stem cell function in the small intestine
Source: Nat Commun. 2026 Jul 29;17:7606. doi: 10.1038/s41467-026-75679-1 (PMC13424321; doi:10.1038/s41467-026-75679-1)
Supplement: Supplementary file 6 — Transparent Peer Review file [file 41467_2026_75679_MOESM6_ESM.pdf]

nature portfolio

Peer Review File

Golgi organization regulates stem cell function in the small intestine

Corresponding Author: Dr Sandra Scharaw

This file contains all reviewer reports in order by version, followed by all author rebuttals in order by version.

Version 0:

Reviewer comments:

Reviewer #2

(Remarks to the Author)
Scharaw et al Nat Comms
Golgi intestinal stem cell

The revised version of the manuscript is much improved over the original paper sent initially to NCB. The authors have addressed many of the points I originally raised, but there are still some issues with the revised manuscript, which I have listed below.

Major point
A major concern relates to the assertion made throughout the paper that lateral Golgi organisation is required for increased secretory trafficking in ISCs and for the optimum signaling between cells. Correlative data would support this, but the only one functional manipulation is used to alter Golgi position, which is depletion of Akap9. The authors have tried as best they can to exclude possible non-Golgi effects of depleting this protein, but nevertheless they cannot be excluded. Another way of altering Golgi position would greatly strengthen paper. Considering the very bold title of 'Golgi organization regulates stem cell function in the small intestine', my feeling is that another way of disrupting Golgi is essential for the author's conclusion to stand.

Other comments
The introduction and discussion are extremely short, which means the paper lacks context and it is difficult to assess how it relates to the state of the art and how novel the new findings are. These sections should be expanded accordingly.

There is a wealth of data provided, which makes the paper hard to follow in places. Better annotation of figure panels would make the data easier to follow.

In terms of the secretory trafficking of EGFR and Lgr5 the method of inducing transport needs to be much more clearly explained in the results text.

The abundance of Akap9 is also quite high in TA and Paneth cells, where the Golgi has a different organisation. It is not clear how Akap9 in ISC cells could lead to a lateral organisation, while it doesn't in other cell types. This should be discussed.

The PDF provided had >5000 pages! Not sure what happened there.

(Remarks on code availability)

Reviewer #4

(Remarks to the Author)
Reviewer 1 raised an original point: "Is the effect of AKAP9 depletion on the number of Golgi elements linked to its role in nucleating microtubule at Golgi membranes? Perform MT nucleation assay in organoids (scramble and AKAP9 depleted)".

The author's response was "the role of AKAP9 in microtubule nucleation at the Golgi has already been well documented across numerous studies". This response as well as the RNA-seq analysis did not directly answer the original question regarding "nucleating microtubule at Golgi membranes".

Reviewer 3's point #3: whether the decreased regeneration was due to mitotic dysfunction in Akap9 knockdown cells, rather than Golgi positioning. The authors provided a Figure that compared Akap9 KO and scramble cells for mitotic cells per crypt. This should be included in the main figures.

Reviewer 3's point #4: "Akap9 add-back experiments are more relevant to address the importance of
Akap9 in preventing/reverting aging through recovering proper Golgi positioning,
reactivating Egf signaling and improving regeneration."
The author's response did not really address this question.

(Remarks on code availability)
N/A

Version 1:

Reviewer comments:

Reviewer #2

(Remarks to the Author)
The authors have done a good job in addressing my comments and I support publication.

(Remarks on code availability)
I had a quick look. It seems to be there.

Reviewer #4

(Remarks to the Author)
The authors addressed the comments raised in the previous review.

(Remarks on code availability)
N/A

Open Access This Peer Review File is licensed under a Creative Commons Attribution 4.0 International License, which permits use, sharing, adaptation, distribution and reproduction in any medium or format, as long as you give appropriate credit to the original author(s) and the source, provide a link to the Creative Commons license, and indicate if changes were made.
In cases where reviewers are anonymous, credit should be given to 'Anonymous Referee' and the source.
The images or other third party material in this Peer Review File are included in the article’s Creative Commons license,

unless indicated otherwise in a credit line to the material. If material is not included in the article's Creative Commons license and your intended use is not permitted by statutory regulation or exceeds the permitted use, you will need to obtain permission directly from the copyright holder.
To view a copy of this license, visit https://creativecommons.org/licenses/by/4.0/

Reviewer #1 (Remarks to the Author):

A. Summary of the key results
The manuscript by Scharaw et al. describes differences in the organization of the Golgi complex and in efficiency of transport of EGFR in intestinal stem cells (ISC) that have one or multiple contacts with Paneth cells. All along the manuscript, the authors use organoids from small-intestinal crypts of young mice (between 3 and 6 months old) or aged mice (22 months old).
The authors showed that EGFR is more expressed (RNA analysis) in ISC than in transient amplifying or in Paneth cells. They use a transport assay for EGFR based on a pulse with high dose of EGF and then release enabling neo-synthesis and transport of EGFR from the endoplasmic reticulum to the cell surface. The organization of the Golgi apparatus is assessed by immunofluorescence and by serial block face scanning electron microscopy. The authors showed that the Golgi apparatus is laterally oriented in ISC.
AKAP9 depletion leads to a lower proportion of crypts cells with lateral Golgi and no difference in EGFR transport efficiency in ISC, in transit amplifying and in Paneth cells.
In addition, the authors show that in crypts from aged mice, EGFR signaling is decreased and similar in the different cell types and that transport to the cell surface of newly synthetized EGFR is reduced while EGFR recycling is increased. Old ISC have only one Golgi element while total Golgi volume is not modified compared to young mice. AKAP9 depletion in old organoids does not further decrease organoid forming capacity compared to aged induced reduction.

B. Originality and significance: if not novel, please include reference
It is an interesting piece of work, bringing new data and concepts
C. Data & methodology: validity of approach, quality of data, quality of presentation
The model used are of high value, the experiments well done and well-presented and the overall quality is excellent
D. Appropriate use of statistics and treatment of uncertainties
I am not an expert, but the data are extensively reported, with mention of replicates, in many cases all values are provided, standard errors showed and statistical tests done
.
E. Conclusions: robustness, validity, reliability
Most of the conclusions are well supported (with some questions bellow)

The reviewer #1 assesses our overall study quality as excellent, well-conducted, well-presented, and with novel concepts, bringing new data and concepts. The reviewer finds that we support most conclusions well, with some questions, to which we added our following new data:

F. Suggested improvements: experiments, data for possible revision
Main Points:
- As only correlation and not direct evidence of the role of Golgi organization on stem cell function is provided, I would suggest to modify the title of the manuscript.

We adjusted the title to: ***Golgi organization regulates stem cell function in the small intestine***

- Is Golgi organization responsible for the stemness/regenerative capacity of ISC or is it the differentiation state (closer to TA/lower Lgr5 expression) that regulates Golgi organization?

We have now addressed this interesting question by inducing Golgi collapse in Akap9-targeted organoids, which led to reduced expression of stem and enteroendocrine markers, while enterocyte marker Alpi was increased, suggesting that Golgi alterations had influenced cell fate choices and stem cell maintenance (Fig. 2F).

Second, to address the other option raised by the Referee (that Golgi organization is influenced by level of differentiation) we treated organoids with CHIR99021 and valproic acid (PMID: 24292484), which resulted in increase of Lgr5+ stem cells in the TA zone. Under this condition, Akap9 mRNA expression was down-regulated (Fig. S3C), which will compromise Golgi organization. Indeed, these Lgr5+ stem cells, which lack mature Paneth cell niche contacts, display collapsed Golgi organization with lost lateral organization (Fig.

S3D). We therefore conclude that the lateral Golgi is regulated by the niche signals in an Akap9-dependent manner and that the Golgi organization is integral to stem cell identity (Fig. 1A,B).

- To which extent are the effects described on EGFR transport valid for other receptors or ligands? Especially, is the efficiency of Lgr5 transport to the cell surface different in the different cell types, which would impact on the amount of Lgr5 at the cell surface and thus dictates their differentiation fate/status? This would be an important observation.

We agree that it is interesting to test if the receptor transport is conserved to other receptors and that Lgr5 is an interesting target to assess. We have therefore established a transport assay for Lgr5 as suggested by the reviewer. Similarly to Egfr synthesis induction, we induced de novo Lgr5 synthesis (See Methods, Fig. S2L). This allowed us to follow the increase in ratio between plasma membrane associated Lgr5 (PM-Lgr5, detected by an antibody recognizing the extracellular domain of Lgr5) and total cellular Lgr5 (detected by an antibody recognizing the intracellular domain of Lgr5). We found that ISC^{MP} transport also Lgr5 receptor more efficiently than ISC^{SP} and TA cells, indicating that the multiple Golgi compartments of ISC^{MP} increase the overall transport of receptors in response to the provided key niche signals (Fig. 1I).

- Have the authors explored the distribution of polarity markers in ISC with single or multiple contacts with Paneth cells?

We now have performed intestinal crypt stainings with the cell polarity markers ZO1 and Par3. These cell polarization markers indicated no differences between ISCs with multiple and single Paneth cell contacts and no differences along the full crypt length (Fig. S1D) suggesting that cell polarisation does not dictate the multiple Golgi organisation in ISC^{MP}.

- Please discuss about the differences of Golgi organization and transport functions already described in polarized and/or differentiated cells.

In the text, we describe the Golgi organisation and transport function in polarised neurons (main text) and of polarised migrating astrocytes and T-cells (discussion).

Other comments:
- Same data represented in Fig1 d (kinetics) and Fig 1h (efficiency at 45 min)

Figure 1d is now Fig 1f. Figure 1h is now Fig 1g. The data in Fig 1f and Fig 1g come from independent experiments and there is no data overlap.

- Is Golgi dispersion responsible for better transport? It would be great to test other means to disperse Golgi and assess transport functions?

To date, we have tested Brefeldin A, Nocodazole, and Latrunculin A as drugs to induce Golgi fragmentation. As reported, all three compounds effectively caused Golgi fragmentation, which in turn inhibited secretory protein trafficking (PMID: 2647301, PMID: 6381504, PMID: 30540523). We acknowledge that it is an interesting avenue to explore the mechanism of fine-controlled Golgi splitting and assess how the level of split Golgi affects ISC EGFR delivery and signal reception in further detail. This is currently addressed as a new project and new publication in the independently started lab of the first author Dr. Scharaw.

- Fig1h and 1i: not easy to assess the number of Golgi elements, provide merge images and/or draw contour of the cells

Figure 1h is now Figure 1g. We provide in Fig. 1a the first of its kind volume EM image of a single stem cell Golgi complex arrangement at super-resolution level. Moreover, we show the E-Cadherin images next to the Golgi images in Fig. 1g. Nevertheless, we have now drawn contour of the cells.

- Fig 2b: perform co-staining with LGR5 (or LGR-eGFP as in Fig1i) to show that AKAP9 expression is higher in ISC (with multiple contacts). Alternatively, describe with more details how ISC MP and ISC SP were identified to generate the graph.

ISC^{MP} in Fig. 2b were identified based on their intercalation between multiple granulated crypt bottom cells (identified from the Bright Field channel) or respectively by their contact with only one for ISC^{SP}. We now added that information to the figure legend and a description in the Method section.

- Why AKAP9 signal is not visible in all cells (as it seems to be expressed even if at lower level)? How normalization has been performed (mean intensity a.u.) ? Does it correspond to fold enrichment?

We have now included a representative image where we increased the intensity of the image, to better highlight Akap9 expression throughout the entire crypt, which displays a much weaker intensity in other cells compared to ISC^{MP} (Fig. 2b). The mean intensity values are the ratio of the single cell IntDen Akap9 intensity/cell area, resulting in arbitrary intensity values. These values are blotted and do not correspond to a fold enrichment. We have clarified this by adding a section on Akap9 quantification in the Method section.

- No description of AKAP9 quantification is provided in the methods section. Please add how it has been performed.

This is now included in the Methods section.

- Fig2d: perform co-staining with acetyl tubulin and AKAP 9 in the crypt

Co-staining of acetylated tubulin and Akap9 in the crypt ISCs is now provided in Fig. 2d lower panel.

- Fig 2e: enrichment of acetyl-Tub in AKAP9 positive cells is not convincing. Please provide other images.

Figure 2e is now Fig. 2d. We now chose more representative images.

- Is the effect of AKAP9 depletion on the number of Golgi elements linked to its role in nucleating microtubule at Golgi membranes? Perform MT nucleation assay in organoids (scramble and AKAP9 depleted)

We thank the reviewer for the suggestion. However, we believe that this experiment is not essential, given that the role of AKAP9 in microtubule nucleation at the Golgi has already been well documented across numerous studies with various cell types and tissues (PMID: 30224411, PMID: 19242490, PMID: 29545805, PMID: 23217741, PMID: 21606206, PMID: 27666745, PMID: 21374596, PMID: 25366663, PMID: 25217626, PMID: 21937724, PMID: 24648492), including in the studies of the author of our study Prof. James Goldenring (PMID: 16356588, PMID: 19073175).

Moreover, we analysed our RNA sequencing data comparing ISC^{MP} and TA progenitor gene expression (see Supplementary Table 1 in the manuscript) for genes known accumulate with Akap9 in a complex to nucleate Golgi-derived microtubules (PMID: 35383336, PMID: 27666745). This demonstrates that these MT-nucleating genes are similarly as Akap9 enriched in ISC^{MP} versus TA progenitor genes (Table 1 for revision). The enrichment in ISC^{MP} happens despite the general trend of secretory pathway genes to be lower expressed in ISC^{MP} compared to TA progenitor cells (see Supplementary Table 4 in the manuscript). This furthermore strengthens our point that Akap9 controls Golgi organization in ISC^{MP} through its role in nucleating microtubule at Golgi membranes.

**Gene ID**	**Mean expression ISC^{MP} (Lgr5^{high})**	**Mean expression TA (Lgr5^{low})**
Akap9	5195.545271	4131.72124
Cdk5rap2	1079.497664	786.1831538
Camsap1	600.5849514	435.5047632

Camsap2	29.9634913	22.3620982
Clasp1	854.418036	792.7704218
Clasp2	671.3129171	588.4048395

Table 1 for revision. Mean RNA sequencing reads of FACS-sorted ISCs (Lgr5^{high}) and Transient Amplifying (TA) progenitor cells isolated from mice. Color scheme indicates low expression in green and high expression in red.

- Fig 3 h and ext data 3d: to show recycling would it be feasible to use fluorescent EGF?

We are following the recycling of the Egf receptor and don't necessarily expect the Egf ligand to also recycle bound to the receptor. Studies, including PMID: 29255092 and PMID: 34032851, demonstrate ligand-unbound Egf receptor recycling, which is controlled by p38 MAPK activation. Interestingly, in ISC and TA cells of the aged intestine p38 MAPK is hyperactivated with age (PMID: 31896747). This suggests that the increased Egfr recycling, which we observe in old ISC and TA (Figure 3h), occurs in a Egf ligand-unbound manner.

To show the recycling of the Egf receptor, we co-stained Egfr with fluorescently labeled transferrin, which is internalized via endosomes and subsequently recycled back to the cell surface. This method is commonly used to visualize receptor recycling (PMID: 12006662, PMID: 19948031).

G. References: appropriate credit to previous work?
As far as I can tell, yes

H. Clarity and context: lucidity of abstract/summary, appropriateness of abstract, introduction and conclusions
Yes, Very clear

Reviewer #2 (Remarks to the Author):

This manuscript describes a role for Golgi apparatus organization and secretory protein trafficking in maintaining the 'stemless' of intestinal stem cells. The intestinal stem cells (ISC) respond to the growth factor EGF while in intestinal crypts, which is mediated by the EGF receptor (EGFR). EGFR, like other plasma membrane proteins, is delivered to the plasma membrane via the Golgi apparatus and conventional secretory pathway. Here, careful analysis indicates a change in Golgi organization in ISC, with an increased number of Golgi stacks (from a typical 1 to 2-3) and a more lateral positioning. This correlates with increased delivery of the EGFR to the plasma membrane. Gene expression analysis suggests the Golgi-microtubule linker protein AKAP9 may be involved, and functional studies support this hypothesis, with depletion of AKAP9 causing reduced number of Golgi stacks, EGFR transport and ability to form intestinal organoids. Analysis of aged versus young animals indicates a similar trend to loss of AKAP9, whose expression is reduced upon ageing. The data together suggest a role for Golgi organization and positioning in stem cell development, which is dependent upon AKAP9 and declines upon ageing.

The importance of Golgi positioning has been studied in various contexts, but to my knowledge not in stem cell biology. The findings are novel, and there is some conceptual novelty i.e. polarized delivery of a signal-receiving receptor to maintain stem cell function in a stem cell niche. The results are likely to be of interest to the cell biology community. However, there were a number of issues that temper my enthusiasm for the study, which are described more fully below:

Reviewer #2 states also that our findings are novel and interesting to the community. Unfortunately, while the reviewer raises important points, we feel that his/her comments miss important biology of the intestinal epithelium. We provide data to the very points raised by the reviewer and made sure to clarify them better.

1.) Additional data is required to show there is a change in trafficking of the EGFR in ISC relative to the other cell types. The EGFR depletion assay requires high EGF for a prolonged period, which is not ideal.

We now demonstrate the changes in trafficking of Egfr in ISC relative to other cell types with several data. First, in Figure 1F, we demonstrate the Egfr trafficking to be more efficient in ISC^{MP} compared to ISC^{SP} and TA cells. This is complemented by the data of Figure 1G, demonstrating that those ISC with multiple Golgi stacks transport Egfr more efficient relative to other cell types. Secondly, in Figure 1H we now demonstrate that the change in Egfr trafficking in ISC^{MP} occurs bi-lateral, unlike in ISC^{SP} where it occurs uni-lateral and in TA cells (seee details explained under Point 2.). Thirdly, we now demonstrate in Figure 1I that also Lgr5 trafficking is specifically changed in ISC compared to TA cells.

Regarding the reviewer's point that the Egfr depletion assay requires high Egf for a prolonged period, we would like to explain in more detail. While we introduce a high EGF pulse for a prolonged time, we continue to only mildly increase the expression levels (1.3-fold, Supplementary Fig2f) of endogenous locus expressed Egf receptor, with no effect on cell or organoid morphology (Supplementary Fig2f). Our transport assay gives the possibility to study endogenous receptor trafficking in a highly quantitative manner by tracking endogenous protein transport with single cell resolution in a 3D tissue context. Other transport assays instead, such as the traditionally used temperature-sensitive VSVG assay and the RUSH assay, are mostly performed in tissue cultured single cells and importantly all assess the transport of overexpressed proteins. These overexpressed transport dynamics might not mimic the endogenous protein transport. Secretion efficiency varies markedly among proteins and can be particularly low for overexpressed proteins (PMID: 9677365, 9104035, 18601052, 2673934). Moreover, the vector-induced overexpression might induce ER stress and lead to unconventional secretion (PMID: 30733486). The first author Dr. Scharaw has herself extensive experience with the ts-VSVG-GFP and RUSH systems through her studies performed in Dr. Rainer Pepperkok's lab at EMBL (PMID: 27872256, 22660414). She, as well as other labs, has experienced possible aggregations of the over-expressed proteins, and problems with protein leakages before the ER release (mentioned for example in 24295307). Together, this raised the absolute need for endogenous protein transport assays, in order to understand physiological relevant protein trafficking.

It also relies on fluorescence microscopy at the place where 2 cells are closely apposed- how much of the EGFR signal is from which cell in this situation?

We feel the Referee overlooks important data and information provided by us. We purposefully chose Egfr and Lgr5 as the receptors for following transport of receptors in ISC, since ISC express the receptors highly, whereas the neighboring PCs do not. This is demonstrated by us on RNA and protein level and the data is included in our manuscript and also discussed. Therefore, there is no concern that our quantification of the Egfr at the lateral membranes would be a mixture from two cells, particularly when we analyze stem cell membranes facing the Paneth cells. In the case where a ISC contacts a Paneth only unilaterally, the receptor transport to the membrane is less efficient – despite the possibility that in such cells the analysis could include some Egfr/Lgr5 from the non-Paneth neighbour. We demonstrate this in Fig. 1d and 1i, where we clearly show the Egfr and Lgr5 ISC enrichment and how it is largely deprived from Paneth cells. We moreover show this on RNA level with our RNA seq data (Supplementary Fig 2a). However, we also carefully revised the text and method sections to minimize the risk of similar misinterpretation by other readers.

2.) If the EGFR is being delivered in a polarized manner to increase signal sensitivity, which is what the model is proposing, then we should see polarized delivery of EGFR to the plasma membrane. This did not appear to be the case.

Indeed, to answer this question, we have now tracked Egfr live transport through the cell upon induced release from the ER with single cell resolution in organoids using time-lapse spinning-disk microscopy, and by developing an image analysis and visualization pipeline. This is now included in the manuscript in Fig. 1h. We find a clear bilateral transport pattern of Egfr in ISC that have bilateral Paneth contacts and therefore bilaterally positioned Golgi stacks. This is in striking contrast to ISC with a single Paneth neighbour, which show unilateral Egfr transport towards the single Paneth cell neighbor and in accordance with their single Golgi stack orientation.

3.) The difference in EGFR transport in ISA compared to TA for example, or ISC with multiple Paneth cell contacts to ISC with few, appears quite small (1 to 1.3 ratio). Is this minor change in EGFR traffic likely to be biologically meaningful?

This is an interesting notion. However, we feel very confident about that ratio difference being meaningful, considering the maximum secretory transport efficiency of cells. Even with boosted overexpression secretion assays such as with the ts-VSVG-GFP, the secretion efficiency of depleted secretory pathway regulator genes versus control gives on average a 1.8 fold difference (PMID: 22660414). Moreover, depletion of well-known secretory pathway core machinery genes, such as genes of the COPII vesicle complex, resulted in a maximum of 1.7 fold deviation from the control (PMID: 22660414). Additionally, similar effect rates as found here, were demonstrated to regulate Egfr secretion specifically (Fig. 6F and H in PMID: 27872256).

The biological relevance of the modest increase in the case of Egfr transport is also influenced by the dynamic nature of Egfr signaling. Internalization of the receptor after ligand binding stipulates a continuous delivery of new receptors, and the low molar abundancy of potent growth factors such as Egf will create a ***competition*** *between receptor presenting cells, conceivably resulting in partial depletion of ligands by the effective Egfr secretors.*

Similarly, the shift from 1 Golgi to 2 smaller Golgi looks like a minor change in organization. I could also not see how this would increase trafficking efficiency. Or is the thinking that fragmenting the ribbon into 2 smaller ones aids the positioning to support secretion towards 2 neighbouring Paneth cells?

We thank the reviewer for the opportunity to clarify our message. Indeed, the working hypothesis is that the formation of multiple Golgi also allows positioning of the multiple stacks towards the neighbouring Paneth cells, e.g. 2 Golgi compartments towards 2 niche Paneth cells. While this makes the stem cell more receptive towards the niche signals coming from the Paneth cells, it is not immediately clear why the split Golgi organisation and lateral positioning also increase the transport on the whole cell level.

We now show that stem cells organize the multiple Golgi compartments in a way that they are parallel oriented towards those Paneth cell neighbours that shared the largest surface area with the ISC (Fig. 1A, Supplementary Fig. 1K, Movie S2). When the Golgi is organized into two compartments with the same total volume as with one compartment, the split compartments deliver Egfr and Lgr5 to the membranes more efficiently, meaning more cargo reaching the plasma membrane at a given time point after release. We hypothesize this to reflect the more efficient vesicle budding from the edges of the Golgi cisterna (PMID: 29148969, PMID: 26311849), in which case multiple Golgi complexes present increased Golgi cisternae edges and thus more vesicles budding sites for secretory transport of cargo than the two edges from a single Golgi complex. Possible other mechanisms include for example vesicle/molecular crowding when secretion is unilateral, or different endocytosis rates. However, given that the Golgi complex is the central organelle of the secretory endomembrane pathway for protein sorting, it is also conceivable that the arrangement into multiple Golgi compartments provides additional benefits for the Golgi function that are not discovered here.

The authors should test whether disrupting the ribbon in other ways also affects ISC EGFR delivery and signal reception.

This is an interesting avenue and commented above in response to the second minor comment of reviewer#1.

5.) The distinction between apical versus lateral for Golgi positioning, as described throughout the paper, is not very clear to me. In most cases, even when the Golgi is described as lateral, it is still apically orientated. Also, the alignment seems to change, and in this case the description of lateral does not seem to be adequate e.g. the alignment can be parallel to the lateral plasma membrane, or it can be tangential to it, regardless of whether the Golgi is relative to the apical-basal axis. These orientations, which I would assume are important, do not seem to have been scored.

We have now developed a fully automated image-based Golgi's orientation analysis to describe whether the Golgi complex is parallel or tangential to the lateral plasma membrane. This approach is described in the Method section and in Supplementary Figure 1l. The results of this analysis are demonstrated in Fig. 2g and Supplementary Fig. 1l and demonstrat that ISC possess a Golgi which is parallel aligned to the lateral membrane whereas in TA progenitor cells Golgi changes to an orientation perpendicular to the lateral plasma membrane, which can be mimicked by Akap9 depletion.

6.) There is a distinction made in the paper between ISC with multiple Paneth cell contacts and those with few. I am struggling to see how these ISC cell types were identified if the ISCs are intercalated with the Paneth cells, with close apposition between the two cell types. How were the contacts identified and therefore the two ISC cell types (low versus high number of contacts)?

We explain our selection criteria in the main text and in the Method section ("modelling of crypt organelles"). However, we want to emphasise here the notion (touched on in the discussion) that almost all ISCs with single Paneth cell contact are found at the niche edge of an intestinal crypt. ISCs contacting one Paneth cell at the edge of the niche have lower likelihood to originate the long-term clone than cells at the center of the niche where ISCs contact multiple Paneth cells (PMID: 35831497, PMID: 24531760). As one-sided niche signals induce differentiation of embryonic stem cells whereas multi-sided signals maintain self-renewal (PMID: 23520113), it is possible that optimization of niche signal receptiveness that is facilitated by Golgi orientation is an important step in many systems.

7.) The model proposes polarized delivery of EGFR as increasing the ability of the ISCs to respond to the EGF signal. However, it seems the total abundance of EGFR also increases in ISC (Fig 1c), so could it not be that they simply express more EGFR, as opposed to any changes in polarized delivery?

We designed our endogenous Egfr and Lgr5 transport assays to account for difference in total Egfr/Lgr5 abundancy by calculating the Egfr/Lgr5 transport efficiency as a ratio between Egfr/Lgr5 at a given time point at the surface over the total cellular Egfr/Lgr5 protein. Therefore, our transport efficiency measurement is normalised to the total Egfr/Lgr5 expression level on a per cell basis. Moreover, we of course agree that there is more Egfr/Lgr5 expression in ISCs (Fig. 1d,i, Supplementary Fig.2a) and for that very reason we chose Egfr/Lgr5 as receptor to study ICS transport efficiency (without interference from Paneth cells).

8.) It is suggested that AKAP depletion preferentially affects the trafficking efficiency in ISC as opposed to the TA cells (Fig 2h). However, depletion of AKAP9 seems to affect the ISC and TA EGFR trafficking, and is not consistent with the model whereby AKAP9 is required to improve the efficiency in ISC only.

We agree that Akap9 affects also transport efficiency of other cell types. However, we now displayed our results in a pairwise comparison that makes it clear that the most striking and only significant drop of transport efficiency is in the ISC^{MP} (Fig. 2h). Given that Akap9 has a role in MT-nucleation from the Golgi, and these Golgi MTs act as cargo transport tracks (PMID: 31984633, 26687990, 15047863), Akap9 depletion will also affect cargo traffikingc from the Golgi in all cell types.

Reviewer #3 (Remarks to the Author):

The present study provides new insights into the regulation of cell signaling in intestinal cells, demonstrating a novel layer of regulation by organelle positioning. The authors investigated the positioning of the Golgi apparatus as a regulatory mechanism of Egf signaling and, consequently, of tissue regeneration. The authors propose that the lateral alignment of the Golgi apparatus in intestinal stem cells enhances the transport efficiency of niche signal receptors, as it positions them closer to Paneth cells, which secrete Egf ligands. This phenomenon seems specific to stem cells, as progenitor/TA cells do not exhibit this phenotype. Mechanistically, the authors identify the anchor protein Akap9 as a key regulator of Golgi alignment in stem cells. Finally, the authors suggest that this mechanism is important in the regeneration of the young intestine, and suggest that its

decay contributes to intestinal aging. The article is clearly written, the figures are complete, and figure legends contain sufficient information for interpretation, including statistical information.
This is indeed an interesting study, as it shows that within the same crypt niche, intestinal cells can differentially respond to cell signaling depending on their intracellular organelle organization. This study exemplifies the importance of investigating the positioning and general biology of organelles as novel determinants of cellular behavior in complex hetero-cellular systems. The authors also present a novel experimental set-up (microscopy-based assay) to evaluate transport efficiency in organoids, a strategy that has the potential to be applied to investigate other signaling pathways. Nevertheless, there are some points that require attention.

Reviewer #3 finds our study interesting and novel, including a novel experimental set-up (microscopy-based assay). The reviewer finds that our study exemplifies the importance of investigating the positioning and general biology of organelles as novel determinants of cellular behavior in complex hetero-cellular systems.

1. Regarding Golgi positioning. It is clearly shown that there are differences in Golgi positioning when comparing crypt bottom cells vs TA cells (extended Fig. 1b). However, within the crypt bottom population, it is less evident whether the lateral positioning of Golgi takes place in SCs (Extended Fig. 1a-c).

We want to draw the attention to Supplementary Fig. 1e, where we show the Golgi complex arrangement in crypt bottom SCs. Moreover, these reasons are exactly why we conducted Volume EM analysis where we demonstrate Golgi arrangement of ISCs at ultrastructure resolution (Fig. 1a-c, Supplementary Fig. 1k,l).

Related to that, although impressive-looking, it is hard to interpret the positioning of Golgi in the different cell types in the EM-based modelling presented in Extended Fig. 2e. Video2 (also impressive), could benefit from adding the color code of the different cell types, and/or freezing frames and adding arrows to highlight the aligning of Golgi in each cell type, etc.

We agree that some images are hard to interpret, and therefore replaced Extended Video 2 with a video with clear cell contours, colour codes to the different cell types and highlighted the aligning of ISC Golgi.

Thus, next to the Golgi stack quantification (Figure 2), the authors should better address Golgi orientation/aligning in each cell, including how the 2 stacks align to two adjacent Paneth cells (as indicated in the manuscript).

This is a very good suggestion, and we have now introduced and included a Golgi alignment score, describing the angle of Golgi in relation to the lateral cell surface towards the adjacent (Paneth) cell plasma membrane. We address this point in the comment 5.) of Reviewer #2.

2. Regarding Akap9 knock-down effect on transport efficiency. Akap9 is highest expressed in the stem cells. The authors propose that Akap9 regulates the positioning of the Golgi in SCs to align it close to PC's contacts as a way of improve transport efficiency. However, knock-down of Akap9 clearly affects transport efficiency in all cell types (Fig. 2h). Does this imply that Akap9 regulates the process independently of Golgi positioning? The authors should address this point.

This notion is also raised by Reviewer #2. We therefore address the comment under point 8) of Reviewer #2.

3. Regarding Akap9 role in regeneration. The authors proposed that Akap9 facilitates stem cell-mediated tissue regeneration by enabling the ISC-specific Golgi stack morphology and high transport efficiency. As mentioned by the authors, Akap9 has also an important role in centrosome regulation and its alteration can lead to increased aneuploidy, impaired cytokinesis and cell cycle arrest (https://www.ncbi.nlm.nih.gov/pmc/articles/PMC194891/). These phenotypes could contribute to a decreased regeneration phenotype of Akap9 knockdown, independently of

Golgi positioning. 50% reduction of Akap9 could still trigger mitotic related phenotypes during regeneration, as regeneration involves active cell division. This needs to be addressed by evaluating if there is any contribution of mitotic dysfunction to the phenotype.

We fully agree that this is an important point. However, two recent studies demonstrate that Akap9 depletion does not affect centrosome function and therefore suggest that cell division remains unaffected. The first is a recent study (PMID: 36796357), which showed that depletion of Akap9-mediated MT-nucleation from the Golgi in neurons had no additive effect on MT nucleation from the centrosome. Similarly, in another study (PMID: 30224411), AKAP9 depletion inhibited Golgi-mediated MT nucleation without impacting centrosomal MT nucleation activity. Based on these findings, the authors concluded that AKAP9 is dispensable for MT nucleation at the centrosome.

Moreover, in the study PMID: 23608191, the authors demonstrate that Akap9 disruption in mice does not affect spindle nucleation or progression of prophase I of meiosis but does inhibit the maturation of Sertoli cells. This further suggests that Akap9 is involved in later stages of cell differentiation rather than cell division.

Nevertheless, to address the reviewer's point, we have now quantified mitotic failure rates in Akap9-depleted organoids. Intestinal stem cells protrude apically during active cell division (PMID: 29897330). We therefore scored the frequency of division events based on the presence of apically protruded cell nuclei. As a result, we did not observe a significant change in the cell division rate in Akap9-depleted compared to Scramble-targeted control cells within intestinal organoids (Figure 1 for revision):

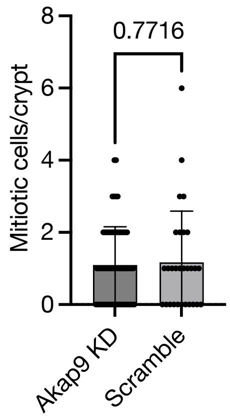

Figure 1 for revision. Crypt domains of Akap9-targeted compared to Scramble-targeted control organoids were analyzed for the number of nuclei in cell division per single imaging Z-Plane of an organoid crypt region. Nuclei were identified based on their Dapi signal and cell surface outlines based on E-Cadherin staining. Mitotic cells were scored as apically protruded cells with dividing nuclei.

4. Regarding Akap9 and Golgi positioning importance in intestinal aging. Related to previous, aneuploidy correlates with aging, and can possibly underlie the phenotype (https://www.nature.com/articles/s41467-018-05258-6). This needs to be taken into consideration when proposing that Akap9 decline underlying aging relates to altered Golgi positioning in SCs. Addressing point 3 (above) could, to some extent, answer to it. Although the aged intestine shares some similarities with Akap9 knock-down in the young intestine, Akap9 add-back experiments are more relevant to address the importance of Akap9 in preventing/reverting aging through recovering proper Golgi positioning, reactivating Egf signaling and improving regeneration.

We fully agree that this is an interesting experiment and the re-introduction or overexpression of AKAP9 in aged intestinal organoids would generate valuable insides into the effect of AKAP9 and mispositioned Golgi with age. However, several studies including by the lab of the first author Dr. Scharaw, suggest that continuous passaging of intestinal organoids derived from aged mice has rejuvenating effects. The genetic modification necessary to re-introduce AKAP9, including proper selection schemes would require several passages of intestinal organoids. It is likely that during the passaging the rejuvenating effect of conditioned media will compromise the suggested experiments and results.

To nevertheless address the reviewer's point that aneuploidy correlates with aging and may underlie the observed Golgi positioning phenotype, we conducted now an analysis based on the study pointed out by the reviewer (https://www.nature.com/articles/s41467-018-05258-6). Specifically, our analysis builds on the observation from that study that Foxm1 expression is repressed with age, which as a result increases aneuploidy and mitotic decline during natural aging.
Therefore, we analyzed our RNA sequencing data comparing SCs of old versus young mice (see Supplementary Table 1 in the manuscript) for changes in Foxm1 expression changes, as well as for the 36 target genes of the Myb-MuvB (MMB)-FOXM1 transcription complex reported in the referenced paper. While we observed Akap9 downregulation in aged ISC^{MP} (Figure S3E in the manuscript), expression of Foxm1 and its target genes did not show an overall downregulation (see Table 2 for revision).
These findings suggest that, in contrast to the octogenarian human dermal fibroblasts described in the referenced study, epithelial ISC^{MP} in aged mouse crypts do not exhibit Foxm1-mediated aneuploidy or mitotic decline. This argues against aneuploidy as the cause of the Golgi phenotype observed in ISC^{MP}.

**Gene ID**	**ISCs Old vs Young**
Akap9	0.822167179
Foxm1	1.202089568
PTTG1	2.282345379
Ccnb2	1.454965893
Aurka	1.349268695
Nek2	1.267596536
Spag5	1.067186711
Ccnb1	1.745658302
Bub1	1.440564364
Cdca8	1.298203989
Kif2c	1.360893154
Kif15	1.132849491
Dlgap5	1.366335609
Bub1b	1.361119505
Cit	1.315337981
Fbxo5	0.990126593
Tpx2	1.244711749
Ndc80	1.020418356
Cep55	0.988473389
Bora	1.198822365
Birc5	1.439455496
Plk1	1.573297081
Kif23	1.159282716
Ncapd2	1.269823867

Cdca2	1.243469378
Cdc25c	1.595812576
Kif18a	1.234702716
Anln	1.219851777
Oip5	0.84449203
Ccna2	1.082152534
Espl1	1.373791027
Mad2l1	1.226048127
Cdk1	1.300664361
Cdc25a	0.928465778
Nuf2	1.368650132
Cdca5	0.960680727

Table 2 for revision. Relative RNA expression fold changes of FACS-sorted ISCs (Lgr5^{high}) cells isolated from old vs. young mice. Expression changes were analyzed based on the reviewer's mentioned study (https://www.nature.com/articles/s41467-018-05258-6) for Akap9, Foxm1 and Foxm1 target genes. Color scheme indicates low expression in green and high expression in red.

Reviewer #2 (Remarks to the Author):

Scharaw et al Nat Comms
Golgi intestinal stem cell

The revised version of the manuscript is much improved over the original paper sent initially to NCB. The authors have addressed many of the points I originally raised, but there are still some issues with the revised manuscript, which I have listed below.

Major point
A major concern relates to the assertion made throughout the paper that lateral Golgi organisation is required for increased secretory trafficking in ISCs and for the optimum signaling between cells. Correlative data would support this, but the only one functional manipulation is used to alter Golgi position, which is depletion of Akap9. The authors have tried as best they can to exclude possible non-Golgi effects of depleting this protein, but nevertheless they cannot be excluded. Another way of altering Golgi position would greatly strengthen paper. Considering the very bold title of 'Golgi organization regulates stem cell function in the small intestine', my feeling is that another way of disrupting Golgi is essential for the author's conclusion to stand.

We thank the reviewer for highlighting the importance of an independent manipulation of Golgi organization. To address this, we performed Akap9 siRNA-mediated knockdown in intestinal cells of Egfr-labelled organoids (Fig. S3C).

C

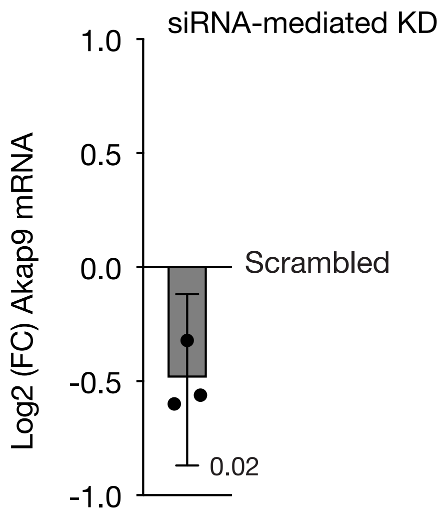

Supplementary Fig. 3C: *RT-qPCR analysis of Akap9 mRNA expression in intestinal organoids transfected with siRNA targeting Akap9 vs. Scramble non-targeting siRNA (organoids from n=3 mice).*

We then pharmacologically rescued Golgi microtubule acetylation using Tubacin, a selective HDAC6 inhibitor that increases microtubule acetylation, and measured Egfr trafficking. First, as expected, Tubacin treatment increased acetylated microtubules at the Golgi complex in both Scrambled and Akap9 depleted crypt bottom intestinal stem cells of organoids (Fig. S3D,E).

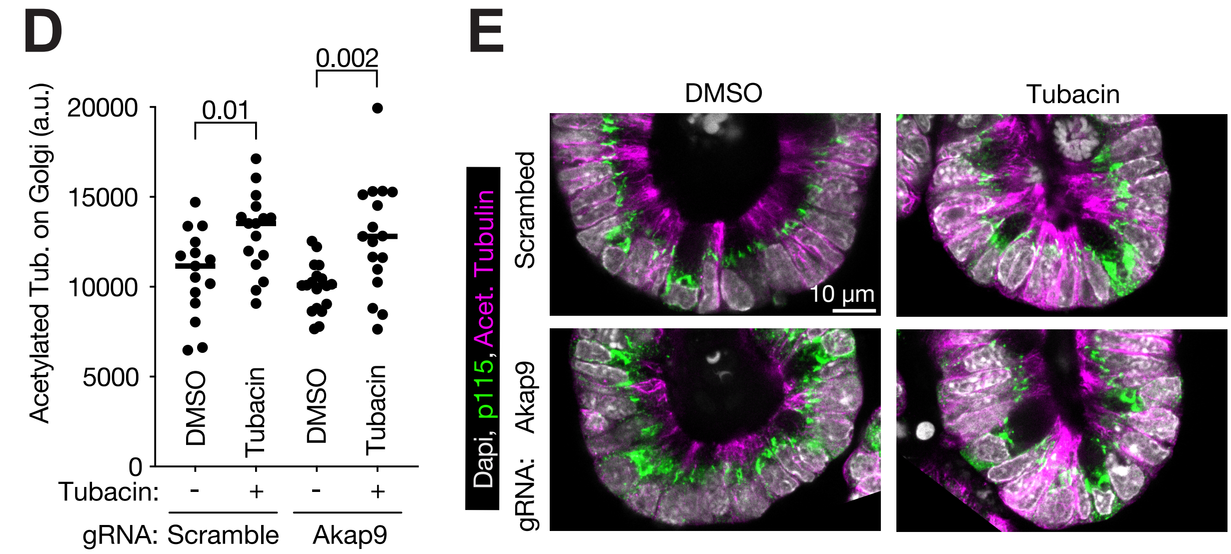

Supplementary Fig. 3: (D) *Quantification and* ***(E)*** *representative images of acetylated tubulin at p115-positive Golgi complex stacks of crypt bottom ISCs in Akap9 Crispr-depleted vs. Scr-targeted organoids treated for 2 hours with 10 µM Tubacin vs. DMSO control. Each data point in (D) represents the mean measured intensity of acetylated tubulin at p115-positive Golgi complexes of ISCs per organoid. (n=3 independent experiments with two-tailed unpaired Student's t-test comparing organoids).*

Importantly, lateral Golgi positioning (Fig. S3F,G) as well as Egfr trafficking (Fig. 2M), which were both impaired in Akap9 depleted cells, were restored upon Tubacin treatment.

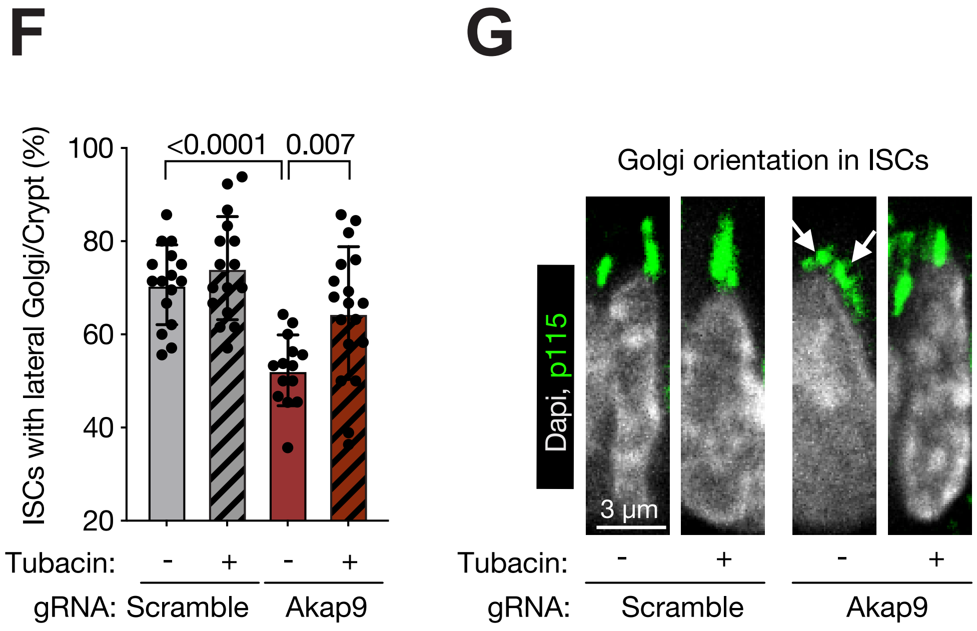

Supplementary Fig. 3: (F) *Quantification and* ***(G)*** *representative images of the percentage of lateral oriented p115-positive Golgi complex stacks of ISCs per crypt in Akap9 Crispr-depleted vs. Scr-targeted control organoids treated for 2 hours with 10 µM Tubacin vs. DMSO Control. Each data point in (F) represents the mean percentage of lateral Golgi stacks of ISCs per organoid. (n=3 independent experiments with two-tailed unpaired Student's t-test comparing organoids).*

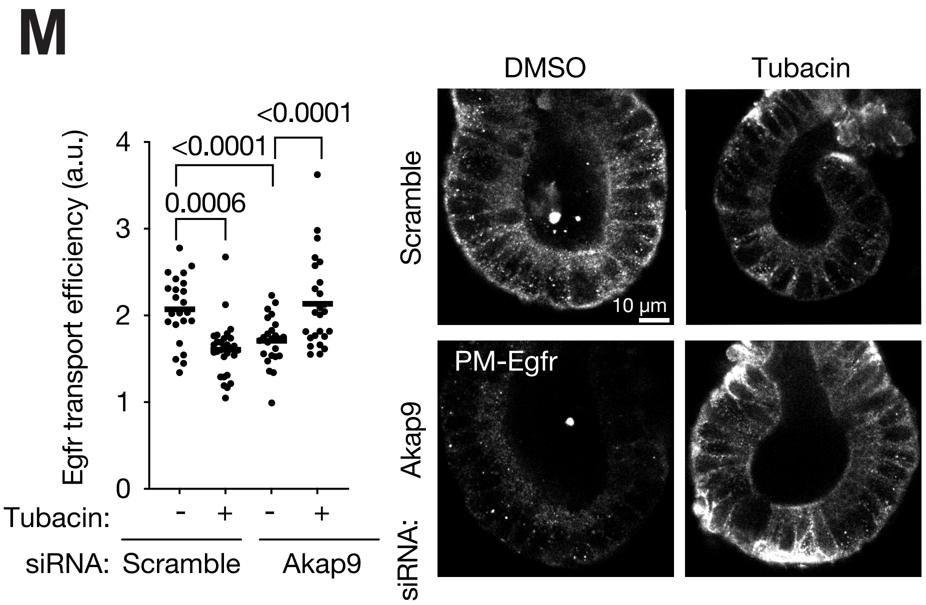

***Fig. 2M:** Egfr transport efficiency at 45 min post-release in Akap9 mRNA down-regulated vs. Scr-targeted control organoids treated with for 2 hours with 10 µM Tubacin vs. DMSO control. Data represent organoids with the mean transport efficiency of single crypt bottom cells per organoid (n=3 mice/condition with two-tailed unpaired Student's t-test comparing the organoids).*

These results indicate that Golgi-associated acetylated microtubule organization itself can compensate for Akap9 loss and is required for efficient receptor trafficking, providing direct functional evidence that supports the role of lateral Golgi organization in efficient stem cell receptor trafficking. We present and discuss this in lines 217-229.

Other comments

The introduction and discussion are extremely short, which means the paper lacks context and it is difficult to assess how it relates to the state of the art and how novel the new findings are. These sections should be expanded accordingly.

We thank the reviewer for the comment. We have now expanded the introduction to a full page (please see lines 56-95), as well as the discussion and included information about the context of the studies and interpretation (lines 289-330). We have also added a section in the discussion, which describes the limitations of the study (please see lines 331-342).

There is a wealth of data provided, which makes the paper hard to follow in places. Better annotation of figure panels would make the data easier to follow.

We have now added annotations to our figures, which make them easier to follow. These include annotations in Fig. 1A and E; in Fig. 2A and G; in Fig. 3A; and in Fig. S1K and L and Fig. S2A.

In terms of the secretory trafficking of EGFR and Lgr5 the method of inducing transport needs to be much more clearly explained in the results text.

We have now explained the Egfr and Lgr5 trafficking assay in detail in the results text (lines 141-150 and 170).

The abundance of Akap9 is also quite high in TA and Paneth cells, where the Golgi has a different organisation. It is not clear how Akap9 in ISC cells could lead to a lateral organisation, while it doesn't in other cell types. This should be discussed.
We have now included the following part to the discussion section (lines 293-299).
"While highly enriched in ISCs, the scaffolding protein Akap9 is also expressed at relatively low levels in TA and Paneth cells. High Akap9 expression in ISCs may exceed a functional thresholding to enable Golgi-based microtubule nucleation and multivalent scaffolding at the Golgi cisternae, pushing Golgi stacks laterally. Instead, lower Akap9 levels likely restrict its activity primarily to centrosomal roles (38), limiting its capacity to reorganize Golgi architecture. Alternatively, Akap9 might act in concert with other proteins specific for ISCs to mediate the peculiar Golgi subcellular localization observed in these cell types."

The PDF provided had >5000 pages! Not sure what happened there.
We have now separated the Supplement Table 1 from the PDF, which was the reason for the length.

Reviewer #4 (Remarks to the Author):

Reviewer 1 raised an original point: "Is the effect of AKAP9 depletion on the number of Golgi elements linked to its role in nucleating microtubule at Golgi membranes? Perform MT nucleation assay in organoids (scramble and AKAP9 depleted)". The author's response was "the role of AKAP9 in microtubule nucleation at the Golgi has already been well documented across numerous studies". This response as well as the RNA-seq analysis did not directly answer the original question regarding "nucleating microtubule at Golgi membranes".
We thank the reviewer for this important point. To directly test whether Akap9 depletion affects microtubule nucleation at Golgi membranes, we performed a nocodazole washout assay and specifically quantified acetylated tubulin within the Golgi region of crypt bottom cells of Scramble and Akap9 Crispr-depleted organoids.
We observed that following 1-hour nocodazole treatment, acetylated tubulin levels were similarly reduced in both control and Akap9-depleted ISCs (~3800 vs ~4200 a.u.). Upon washout, control cells showed robust microtubule re-establishment at the Golgi, whereas Akap9-depleted cells displayed significantly impaired recovery (Scrambled: ~9300 vs. Akap9 KD: ~6800).

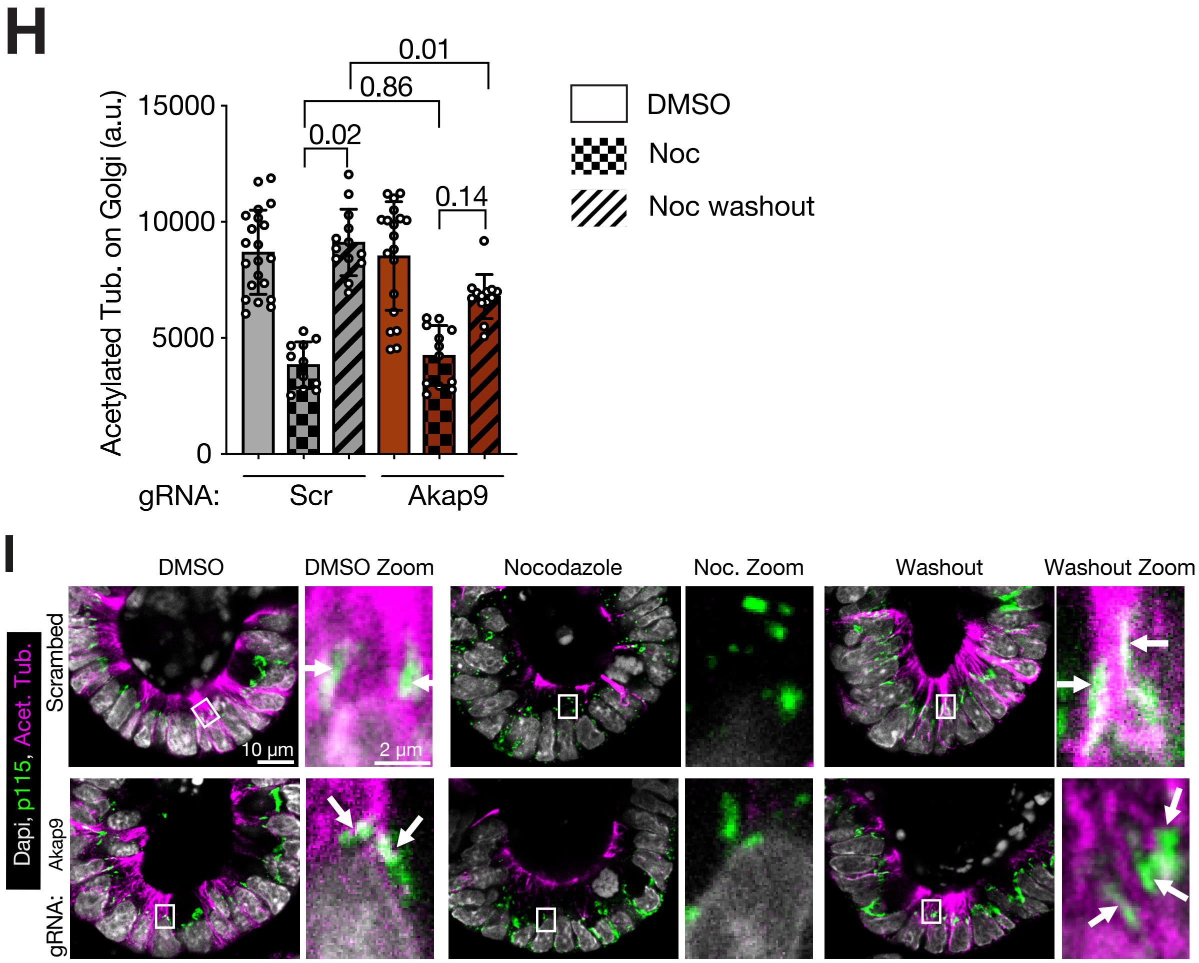

Fig. 2: ***(H)*** *Quantification and* ***(I)*** *representative images of acetylated tubulin re-nucleation at p115-positive Golgi complex stacks of crypt bottom ISC cells in Akap9 Crispr-depleted vs. Scr-targeted control organoids. Organoids were treated for 1 hour with 10 µM Nocodazole, followed by a 2 hours washout. Each data point in* ***(H)*** *represents the mean measured intensity of acetylated tubulin at p115-positive Golgi complexes of ISCs per organoid. (n=3 independent experiments with two-tailed unpaired Student's t-test comparing organoid averages per experiment).*

These results provide direct functional evidence that Akap9 is required for efficient microtubule re-nucleation at Golgi membranes. This defect in Golgi-associated microtubule re-establishment provides a mechanistic explanation for the loss of lateral Golgi polarization in our system, supporting a model in which Akap9-dependent microtubule nucleation regulates lateral Golgi organization through its role in microtubule nucleation at Golgi membranes. This analysis has been incorporated into the revised manuscript (Fig. 2H,I and lines 196-206).

Reviewer 3's point #3: whether the decreased regeneration was due to mitotic dysfunction in Akap9 knockdown cells, rather than Golgi positioning. The authors provided a Figure that compared Akap9 KO and scramble cells for mitotic cells per crypt. This should be included in the main figures.

L

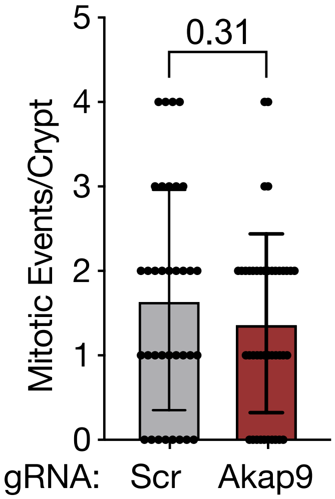

Fig. 2L: *Crypt domains of Akap9 Crispr-targeted vs. Scramble-targeted control organoids were analyzed for the number of nuclei in cell division per single imaging z-plane of an organoid crypt region. Nuclei were identified based on their Dapi signal and cell surface outlines based on bright field images. Mitotic cells were scored as apically protruded cells with dividing nuclei (n=4 experimental replicates).*

We now included the data into the revised manuscript (Fig. 2L, line 214-215).

Reviewer 3's point #4: "Akap9 add-back experiments are more relevant to address the importance of
Akap9 in preventing/reverting aging through recovering proper Golgi positioning, reactivating Egf signaling and improving regeneration."
The author's response did not really address this question.
As we outlined already previously in our response, we fully agree that this is an interesting experiment and the re-introduction or overexpression of Akap9 in aged intestinal organoids woul d generate valuable insides into the effect of Akap9 and mispositioned Golgi with age. However, we again want to highlight that continuous passaging of intestinal organoids derived from aged m ice has rejuvenating effects. The genetic modification necessary to re-introduce Akap9, includin g proper selection schemes of the re-introduced Akap9 requires several passages of intestinal or ganoids and we are therefore not able to conduct the suggested experiments since they would n ot be reliable.

[REDACTED]

We have now included a discussion of these limitations in the revised manuscript (lines 331-342) and clarified that our current findings are correlative (line 262, Fig. 3 headline), showing that aged stem cells exhibit impaired Golgi morphology and decreased receptor transport (Fig. 3 B,D).
